# Deficiency of *CHAMP1*, a gene related to intellectual disability, causes impaired neuronal development and a mild behavioural phenotype

**DOI:** 10.1093/braincomms/fcac220

**Published:** 2022-08-30

**Authors:** Masayoshi Nagai, Kenji Iemura, Takako Kikkawa, Sharmin Naher, Satoko Hattori, Hideo Hagihara, Koh-ichi Nagata, Hayato Anzawa, Risa Kugisaki, Hideki Wanibuchi, Takaya Abe, Kenichi Inoue, Kengo Kinoshita, Tsuyoshi Miyakawa, Noriko Osumi, Kozo Tanaka

**Affiliations:** Department of Molecular Oncology, Institute of Development, Aging and Cancer (IDAC), Tohoku University, Sendai, Miyagi 980-8575, Japan; Department of Molecular Oncology, Institute of Development, Aging and Cancer (IDAC), Tohoku University, Sendai, Miyagi 980-8575, Japan; Department of Developmental Neuroscience, United Centers for Advanced Research and Translational Medicine (ART), Tohoku University Graduate School of Medicine, Sendai, Miyagi 980-8575, Japan; Department of Developmental Neuroscience, Tohoku University Graduate School of Life Sciences, Sendai, Miyagi 980-8575, Japan; Division of Systems Medical Science, Institute for Comprehensive Medical Science (ICMS), Fujita Health University, Toyoake, Aichi 470-1192, Japan; Division of Systems Medical Science, Institute for Comprehensive Medical Science (ICMS), Fujita Health University, Toyoake, Aichi 470-1192, Japan; Department of Molecular Neurobiology, Institute of Developmental Research, Aichi Developmental Disability Center, Kasugai, Aichi 480-0392, Japan; Department of Neurochemistry, Nagoya University Graduate School of Medicine, Nagoya, Aichi 466-8550, Japan; Department of Applied Information Sciences, Graduate School of Information Sciences, Tohoku University, Sendai 980-8579, Japan; Department of Molecular Oncology, Institute of Development, Aging and Cancer (IDAC), Tohoku University, Sendai, Miyagi 980-8575, Japan; Department of Molecular Pathology, Osaka City University Graduate School of Medicine, Osaka 545-8585, Japan; Laboratory for Animal Resources and Genetic Engineering, RIKEN Center for Biosystems Dynamics Research, Kobe, Hyogo 650-0047, Japan; Laboratory for Animal Resources and Genetic Engineering, RIKEN Center for Biosystems Dynamics Research, Kobe, Hyogo 650-0047, Japan; Department of Applied Information Sciences, Graduate School of Information Sciences, Tohoku University, Sendai 980-8579, Japan; Division of Integrated Genomics, Tohoku Medical Megabank Organization, Tohoku University, Sendai, 980-8573, Japan; Department of In Silico Analysis, Institute of Development, Aging and Cancer, Tohoku University, Sendai, 980-8575, Japan; Division of Systems Medical Science, Institute for Comprehensive Medical Science (ICMS), Fujita Health University, Toyoake, Aichi 470-1192, Japan; Department of Developmental Neuroscience, United Centers for Advanced Research and Translational Medicine (ART), Tohoku University Graduate School of Medicine, Sendai, Miyagi 980-8575, Japan; Department of Molecular Oncology, Institute of Development, Aging and Cancer (IDAC), Tohoku University, Sendai, Miyagi 980-8575, Japan

**Keywords:** *CHAMP1*, mouse model, neuronal development, intellectual disability, behavioural tests

## Abstract

*CHAMP1* is a gene associated with intellectual disability, which was originally identified as being involved in the maintenance of kinetochore–microtubule attachment. To explore the neuronal defects caused by *CHAMP1* deficiency, we established mice that lack *CHAMP1*. Mice that are homozygous knockout for *CHAMP1* were slightly smaller than wild-type mice and died soon after birth on pure C57BL/6J background. Although gross anatomical defects were not found in *CHAMP1*^−/−^ mouse brains, mitotic cells were increased in the cerebral cortex. Neuronal differentiation was delayed in *CHAMP1*^−/−^ neural stem cells *in vitro*, which was also suggested *in vivo* by CHAMP1 knockdown. In a behavioural test battery, adult *CHAMP1* heterozygous knockout mice showed mild memory defects, altered social interaction, and depression-like behaviours. In transcriptomic analysis, genes related to neurotransmitter transport and neurodevelopmental disorder were downregulated in embryonic *CHAMP1*^−/−^ brains. These results suggest that CHAMP1 plays a role in neuronal development, and *CHAMP1*-deficient mice resemble some aspects of individuals with *CHAMP1* mutations.

## Teaser

Nagai *et al.* report the first characterization of a knockout mouse of *CHAMP1*, a gene mutated in individuals with intellectual disability. *CHAMP1* deficiency delayed neuronal development and *CHAMP1* heterozygous knockout mice exhibited mild memory defects, altered social interaction, and depression-like behaviours, resembling some aspects of the human phenotype.

## Graphical Abstract

**Graphical abstract:**
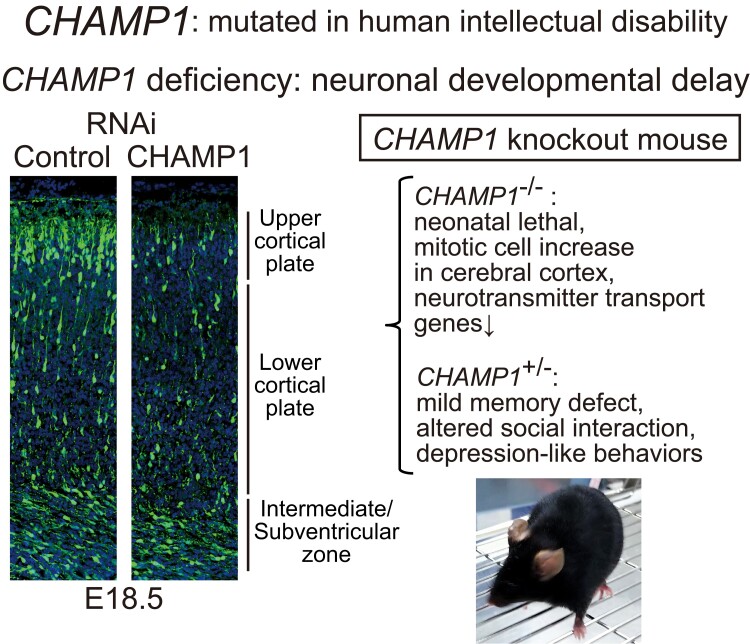

## Introduction

Intellectual disability (ID) is a common developmental disorder that affects 2–3% of the general population.^1,2^ As a clinical entity, ID is included in neurodevelopmental disorders (NDDs) together with autism spectrum disorder (ASD) and attention deficit hyperactivity disorder (ADHD). In the *Diagnostic and Statistical Manual of Mental Disorders, 5th Edition* (DSM-5), ID is defined as NDD that begins in childhood and is characterized by intellectual difficulties as well as difficulties in conceptual, social and practical areas of living.^3^ Both environmental stress factors, such as poor nutrition and infection, and mutations in genes cause ID.^4^ Through large-scale studies, a large number of mutated genes implicated in ID have been identified in nearly half of the cases, although mutations of each gene occur in a small subset of cases.^5,6^ How such a diverse spectrum of genes cause ID is largely unknown, and effective therapeutic interventions are lacking.

**Figure 1:**
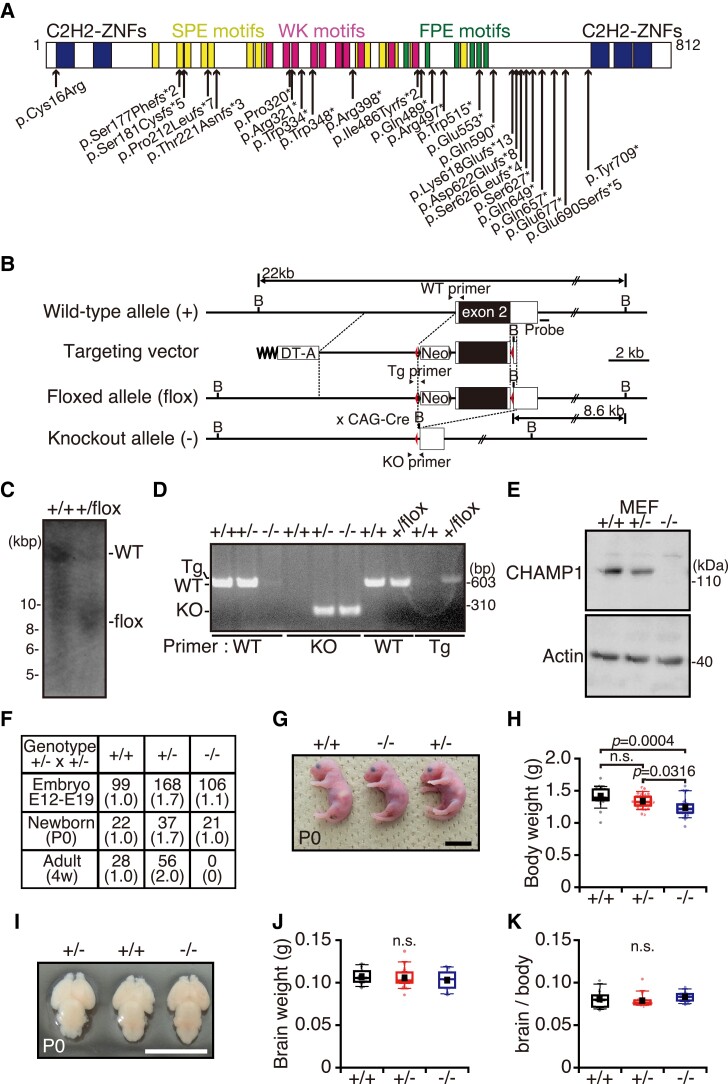
***CHAMP1* ^−/−^ mice show a newborn lethal defect.** (**A**) CHAMP1 mutations found in individuals with ID. A schematic structure of CHAMP1 and its ID-associated *de novo* mutations are shown. fs, frameshift; *, premature stop codon. (**B**) A schematic of construction of the *CHAMP1*-knockout allele. Positions of the probes for Southern blotting and PCR primers are indicated. Red triangle shows positions of the *lox-P* sites. Neo, neomycin-resistance gene; DT-A, diphtheria toxin A gene; B, BamHI site. (**C**) Southern blot analysis of genomic DNA prepared from *CHAMP1*^+/+^ and *CHAMP1*^+/*flox*^ mice with the probe shown in (**B**). **(D**) Genotyping PCR analysis of *CHAMP1*^+/+^, *CHAMP1*^+/*flox*^, *CHAMP1*^+*/−*^, and *CHAMP1*^−/−^ mice using the primer sets shown in (**B**). (**E**) IB analysis of lysates prepared from MEFs isolated from *CHAMP1*^+/+^, *CHAMP1*^+*/−*^, and *CHAMP1*^−/−^ mice using antibodies as indicated. (**F**) Number and ratio of *CHAMP1*^+/+^, *CHAMP1*^+*/−*^, and *CHAMP1*^−/−^ mice from crosses of C57BL/6J *CHAMP1*^+*/−*^ mice. (**G**) Representative picture of newborn *CHAMP1*^+/+^ (left), *CHAMP1*^+*/−*^ (right), and *CHAMP1*^−/−^ (middle) mice. Scale bar: 1 cm. (**H**) Comparison of the body weight of newborn mice (*CHAMP1*^+/+^; *n* = 19, *CHAMP1*^+*/−*^; *n* = 36, *CHAMP1*^−/−^; *n* = 19). (**I**) Representative pictures of newborn *CHAMP1*^+/+^ (middle), *CHAMP1*^+*/−*^ (left), and *CHAMP1*^−/−^ (right) mouse brains. Scale bar: 1 cm. (**J, K**) Absolute brain weight (**J**) and ratios of brain weight to body weight (**K**) of newborn mice (*CHAMP1*^+/+^; *n* = 8, *CHAMP1*^+*/−*^; *n* = 14, *CHAMP1*^−/−^; *n* = 8). For box plots in (**H, J, K**), the bottom and top of the box show the lower and upper quartile values, respectively. The mean is indicated with a filled square and the median is indicated with a bar in the box. The bottom and top of the whiskers denote the 10th and 90th percentiles, respectively. *P* values were determined by Tukey–Kramer test. *n.s.*, not statistically significant.

*CHAMP1* is one of the genes mutated in individuals with ID.^7–9^ A recent report showed that disorders associated with *CHAMP1* mutations also include ASD and ADHD phenotypes.^10^ We originally reported that this gene product is involved in the maintenance of kinetochore–microtubule attachment on the spindle during mitosis and designated the molecule as CAMP (chromosome alignment-maintaining phosphoprotein),^11^ registered as *CHAMP1* in Human Gene Nomenclature Database. CHAMP1 (CAMP) is conserved in vertebrates, and human CHAMP1 is an 812 aa protein composed of a large intrinsically disordered region with zinc-finger domains at each end (Fig. 1A). The middle region contains several repeat motifs and it is phosphorylated at many serine residues, especially in mitosis, by CDK1 (cyclin-dependent kinase 1).^11^ CHAMP1 localizes to the nucleus in interphase and to chromosomes and the spindle during mitosis.^11^ In mice, CHAMP1 expression is detected in brain, thymus, testis and ovary tissues.^8^ In individuals with ID, most of the mutations identified in *CHAMP1* are either nonsense or frameshift *de novo* mutations that result in the formation of truncated proteins lacking the C-terminal region to varying degrees (Fig. 1A).^7–10,12–15^ We reported that these *C*-terminally truncated CHAMP1 mutant proteins cannot properly localize to the chromatin in interphase nor to chromosomes in mitosis.^8^ Some individuals with *CHAMP1* mutations show other symptoms such as microcephaly, facial dysmorphism and convulsions.^7–10^ It has been reported that microdeletion of the 13q34 locus that includes *CHAMP1* also causes a similar phenotype,^13,16,17^ which is sometimes inherited. Intriguingly, the gene encoding pogo transposable element-derived protein with zinc-finger domain (POGZ), an interacting partner of CHAMP1, is also mutated in individuals with ID, as well as in individuals with ASD,^18–21^ causing a variety of phenotype known as White-Sutton syndrome.^22^ The *POGZ* mutations found in ID are either nonsense or frameshift *de novo* mutations similar to *CHAMP1* mutations. We found that CHAMP1 and POGZ interact with their *C*-terminal regions,^8^ suggesting that the interaction between CHAMP1 and POGZ is lost in mutants of both proteins, which may be related to the aetiology of ID.

Mouse models have contributed to the understanding of the pathophysiology of ID and ASD.^4^ To explore the neuronal functions of CHAMP1 related to ID, we generated a *CHAMP1* knockout mouse. We found that *CHAMP1* homozygous knockout mice die soon after birth. Although brain structure of *CHAMP1*^−/−^ mice was grossly normal, *CHAMP1*^−/−^ neural stem cells (NSCs) showed a delay in neuronal differentiation *in vitro*, which was supported by CHAMP1 knockdown *in vivo*. In a behavioural test battery, *CHAMP1* heterozygous knockout mice exhibited mild memory defects, altered social interaction and depression-like behaviours. Transcriptomic analysis revealed downregulation of neurotransmitter transport and NDD-related genes. Our study suggests that CHAMP1 plays a role in neuronal development, and *CHAMP1* knockout mice contribute to understanding the pathophysiology of disorders seen in individuals with *CHAMP1* mutations.

## Materials and methods

### Mice

*CHAMP1* mutant mice (Accession No. CDB 1106K: https://large.riken.jp/distribution/mutant-list.html) were established as described (https://large.riken.jp/protocol/). To construct a targeting vector, a neomycin resistant gene, frt sequences and lox-P sites were inserted into the BAC clone containing Exon 2 of the *CHAMP1* gene (RP24-377P6, BACPAC resources) (Fig. 1B). TT2 embryonic stem cells^23^ were electroporated with the linearized targeting vector and selected with G418 (Thermo Fisher Scientific). The homologous recombinant ES clones were identified by PCR and Southern blotting and were used to generate chimeric mice. The chimeric mice were crossed with C57BL/6J (wild-type) mice to obtain *CHAMP1^+/flox^* mice. The recombination event was confirmed by Southern blot analysis of BamHI-digested genomic DNA, which was performed at Phoenix Bio Co., Ltd (Japan). *CHAMP1^+/−^* mice were generated by breeding *CHAMP1^+/flox^* male mice with CAG-Cre female mice.^24^  *CHAMP1* knockout mice were backcrossed into C57BL/6J for at least five generations. We bred the animals in groups of one or two under specific pathogen-free conditions (*ad libitum* access to food and water, 12:12 light:dark cycle, light on at 08:00 am). Mice were tagged using ear punch and randomly assigned to each experiment. All experimental procedures conformed to ‘Regulations for Animal Experiments and Related Activities at Tohoku University’ and ‘Guidelines for Conducting Animal Experiments at RIKEN Kobe Branch’ and were reviewed by the Institutional Laboratory Animal Care and Use Committee of Tohoku University and the Institutional Animal Care and Use Committee of RIKEN Kobe Branch and finally approved by the President of Tohoku University (2019AcA-024) and RIKEN Kobe Branch (A2001-03), and the same procedure was performed in Fujita Health University (AP16016).

### Antibodies and PCR primers

Anti-CHAMP1 polyclonal antibody was purified using antigen-conjugated HiTrap NHS-activated HP Columns (GE Healthcare) from antiserum of rabbits immunized against mouse CHAMP1 aa515–646 (UNITECH) and used for immunofluorescence (IF) and immunoblot (IB) at 1:1000. Primary antibodies used in IF were as follows: mouse anti-Nestin (1:1000, Abcam), mouse anti-Tuj1 (βIII tubulin; 1:1000, BioLegend), rabbit anti-Pax6 (1:300, BioLegend), rabbit anti-Pax6 (1:1000, MBL), rabbit anti-Cux1 (1:1000, Santa Cruz Biotechnology), rat anti-Ctip2 (1:1000, Abcam), chicken anti-green fluorescent protein (GFP) (1:1000, Abcam), mouse anti-phospho-histone H3 Ser10 (1:1000, Cell Signalling), rabbit anti-PCNT (pericentrin; 1:1000, Abcam), rabbit anti-Ki67 (1:1000, Novusbio), mouse anti-glial fibrillary acidic protein (GFAP) (1:1000, Thermo Fisher Scientific), rabbit anti-Map2 (1:1000, BioLegend), mouse anti-Ankyrin-G (1:1000, Santa Cruz Biotechnology), and rabbit anti-POGZ (1:300).^25^ Polyclonal goat antibody against actin (Santa Cruz Biotechnology) was used in IB at 1:1000. Alexa Fluor 488-rabbit (Thermo Fisher Scientific), Alexa Fluor 568-mouse (Thermo Fisher Scientific), and Alexa Fluor 488-rat (Jackson Immunoresearch Laboratories) were used as secondary antibodies in IF at 1:1000. Alexa Fluor 488-chicken (1:500; Jackson Immunoresearch Laboratories), Cy3-anti-rabbit IgG (1:500; Jackson Immunoresearch Laboratories), Cy3-anti-rat IgG (1:500; Jackson Immunoresearch Laboratories) were used as secondary antibodies in IF at 1:500. Rabbit IgG-HRP (Santa Cruz Biotechnology) and goat-IgG-HRP (Santa Cruz Biotechnology) were used as secondary antibodies in IB at 1:1000. To stain DNA, 4′,6-diamidino-2-phenylindole (DAPI) was used at 1 μg/ml. The following primers were used for genotyping PCR: forward primer: 5′- CCTGAGAGGCCCCAAATAAAACTCCAAGTG - 3′; reverse primers: 5′- CTCGGTGTCTTTGCTCGGCTGCTCTTTCGG - 3′ (wild-type allele), 5′- TGAGCCCAGAAAGCGAAGGAGCAAAG −3′ (floxed allele), and 5′- CAGACCAGGATACACCACTACACACATACT - 3′ (knockout allele).

### Cell culture

Mouse embryonic fibroblasts (MEFs) were isolated from embryonic (E) Day 14.5 embryos and maintained at 37°C in 5% CO_2_ atmosphere in Dulbecco’s modified Eagle’s medium (DMEM, Nacalai Tesque) supplemented with 10% foetal bovine serum and 20 mM 4-(2-hydroxyethyl)-1-piperazineethanesulfonic acid. NSCs were cultured essentially as previously described.^26^ Briefly, brains from E14.5 mice were triturated in DMEM/F12, GlutaMAX supplement (Thermo Fisher Scientific) and cultured for 4 days in DMEM/F12, GlutaMAX supplement, containing N2 supplement (R & D Systems) added with 20 ng/ml EGF (Merck) and 20 ng/ml FGF2 (Merck) on dishes precoated with 15 μg/ml poly-L-ornithine (Merck) and 1 μg/ml fibronectin (Merck). Cells were then detached in DMEM/F12, GlutaMAX supplement and re-plated on dishes precoated as above, and cultured for 5 days in DMEM/F12, GlutaMAX supplement, containing N2 supplement without EGF and FGF2. In Supplementary Fig. 7, NSCs were cultured using neurosphere culture method.^27^ Briefly, cells were dissociated with neuron dissociation solutions (FUJIFILM Wako) according to the manufacturer’s instructions from the cortex in the E13.5 mouse brain and cultured for 4 days in the NSC culture medium as above on Costar Ultra-low attachment plate (Corning). Neurospheres were suspended and put on the dishes precoated with 15 μg/ml poly-L-ornithine and 1 μg/ml fibronectin. After 24 h, the neurospheres were transfected with a control or *CHAMP1* siRNA using RNAiMAX (Thermo Fisher Scientific).. The sequences of siRNAs are as follows: *CHAMP1* siRNA, AUAUGUCUGACGCUACCUUCGGUGA; control siRNA, UUCCUCUCCACGCGCAGUACAUUUA (Stealth; Thermo Fisher Scientific). Neurospheres were then differentiated for 5 days in the absence of EGF and FGF2 as described above. Primary cortical neurons were dissociated with neuron dissociation solutions (FUJIFILM Wako) according to the manufacturer’s instructions. Dissociated neurons were seeded on coverslips or dishes coated with 10 μg/ml poly-D-lysine (PDL; Merck) and 2 μg/ml Cultrex Mouse Laminin I (Trevigen) cultured in Neurobasal medium (Thermo Fisher Scientific) containing N21-MAX Media supplement (R & D Systems) and 0.5 mM L-glutamine (Nacalai Tesque).

### Immunofluorescence staining and microscopic observation

Immunohistochemistry of frozen sections was performed according to our previous report.^28^ All samples were fixed in 1% paraformaldehyde (PFA) in phosphate-buffered saline (PBS; 137 mM NaCl, 2.7 mM KCl, 10 mM Na_2_HPO_4_ and 1.8 mM KH_2_PO_4_, pH 7.4) for 12 h. Brains were cryoprotected in 10% and then 20% sucrose in PBS for 12 h each at 4°C and were frozen with Tissue-Tek O.C.T. compound (Sakura Finetek Japan). Coronal or sagittal sections were cut for 14 μm thickness using cryostat CM3050S (Leica Biosystems) and mounted onto MAS-coated glass slides (Matsunami Glass). Brain sections were masked with 3% albumin, from bovine serum, Fraction V pH7.0 (BSA, Fujifilm Wako) in TBST (403 mM Tris, pH 7.5, 137 mM NaCl, 27 mM KCl, and 0.1% Triton-X100) and were incubated with primary antibodies in 3% BSA/TBST or Can Get Signal immunostain solution A (TOYOBO) for 12 h at 4°C. After washes with TBST, sections were incubated with secondary antibodies in 3% BSA/TBST for 1 h at room temperature and were mounted with ProLong Gold (Thermo Fisher Scientific). Images were captured by a confocal laser microscope LSM800 (Zeiss) and BZ-9000 microscope (Keyence) using ×4, 0.20 numerical aperture (NA) Plan Apochromat air objective lens (Nikon) or ×10, 0.45 NA Plan Apochromat air objective lens (Nikon). *Z*-image stacks were captured in 0.2 μm increments on an IX-83 inverted microscope (Olympus) using ×60, 1.35 NA UPlan SApochromat oil objective lens (Olympus). Deconvolution was performed when necessary. Image stacks were projected and saved as TIFF files. Each experiment was successfully repeated at least three times.

### Nissl staining

Cryosections after post-fixation with 4% PFA in PBS were washed in TBST, and incubated with NeuroTrace™ 500/525 Green Fluorescent Nissl Stain (1:300, Molecular Probes) in PBS for 30 min. After washing with TBST, the sections were mounted with VECTASHIELD antifade mounting medium (Vector Laboratories Inc).

### *In situ* hybridization

*In situ* hybridization for frozen sections was performed as previously described.^29,30^ To obtain templates for cDNA fragment, full-length mouse *CHAMP1* cDNA was amplified by PCR from a cDNA clone (mKIAA1802) in the ROUGE database (http://www.kazusa.or.jp/rouge/) and was inserted into EcoR1 and Xho1 sites in pcDNA3.1Zeo (−) expression vector (Thermo Fisher Scientific). A riboprobe was obtained from a *CHAMP1* cDNA fragment (1542–2409 bp), which was cloned into a pBluescript IISK (−) (Agilent Technologies). *POGZ* riboprobe was obtained from a *POGZ* cDNA fragment (1–1000 bp), which was amplified from a cDNA clone (MMM1013-202761079, Horizon Discovery) and cloned into a pBluescript IISK (−).

### Western blotting

Brains and MEFs were lysed in TNE-N buffer (1% NP-40, 150 mM NaCl, 20 mM Tris HCl, pH 7.5, and 2 mM EDTA). Protein concentration was measured by the Bio-Rad Protein assay kit (Bio-Rad). Extracted proteins were boiled for 5 min with 4× NuPAGE LDS (lithium dodecyl sulphate) sample buffer (Thermo Fisher Scientific). Proteins were separated by NuPAGE sodium dodecyl sulphate (SDS)-polyacrylamide gel electrophoresis (PAGE) Gel System (Thermo Fisher Scientific), electroblotted onto a polyvinylidene difluoride membrane (PVDF; Amersham Hybond-P, GE Healthcare). PVDF membrane was masked with 3% non-fat dry milk in TBST (150 mM NaCl, 0.1% Tween20, 25 mM Tris HCl pH7.5) for 30 min at room temperature, incubated with primary antibodies in Can Get Signal IB enhancer Solution I (TOYOBO) for 12 h at 4°C, washed with TBST and incubated with secondary antibodies in 3% BSA for 1 h in TBST at room temperature. After final washes, proteins were visualized using enhanced chemiluminescence, according to the manufacturer’s instructions. Chemiluminescence signals were detected using LAS4000mini Lumino Image Analyzer (GE Healthcare).

### *In utero* electroporation

*In utero* electroporation of E14.5 C57BL/6J mouse cortex was performed as previously described with a minor modification.^31,32^ Specific siRNA for *CHAMP1* or control, which is the same as the one used in *in vitro* culture, and *pCAG-EGFP* (kindly gifted from Prof. Tetsuichiro Saito, Chiba University, Japan) were injected into the lateral ventricle of the brain at concentrations of 2.0 μg/μl and 0.5 μg/μl, respectively. Injections were carried out using a glass capillary with the electric microinjector system (Narishige). The electroporation into progenitor cells in the ventricular zone (VZ) was performed with five pulses of 40 V using a square wave pulse electroporator (BEX). Electroporated embryos were fixed at E16.5 and E18.5.

### Terminal deoxynucleotidyl transferase dUTP nick end labelling staining

The *in situ* cell death detection kit (Merck) was used to detect apoptotic cells according to the manufacturer’s protocol. Briefly, the sections were fixed in 4% PFA for 15 min at room temperature. The sections were incubated in 0.1% Triton X-100 for 2 min on ice. Transferase dUTP nick end labelling (TUNEL) mixture was added to brain sections and incubated in a humidified chamber for 60 min at 37°C.

### Golgi-Cox staining and spine density analysis

Golgi-Cox staining was performed according to the manufacturer’s protocol of an FD Rapid Golgi Stain Kit (FD Neurotechnologies). Briefly, the unfixed brains were immersed in specific impregnation solution containing Solution A and B (1:1) in darkness at room temperature for 14 days and then incubated in solution C at 4°C in darkness for 3 days. After freezing with dry ice, the brains were sliced into 100 μm pieces using a vibratome (Leica VT1200; Leica Biosystems) and mounted on gelatin-coated slides with solution C. After drying at room temperature, the sections were stained with a mixed solution consisting of Solution C, Solution D and Milli-Q water at 1:1:2 for 5 min. After sections were rinsed in Milli-Q water, each section was dehydrated in graded alcohol solutions, cleared in xylene, and coverslipped. Spines were judged mushroom if the diametre of the head was much greater than the diametre of the neck, according to the previously described criteria.^33^

### Sholl analysis

Image stacks of neurons differentiated from NSCs for 5 days immunostained with a Tuj1 antibody were traced and reconstructed as 2D binarized representations using ImageJ (National Institute of Health, http://rsb.info.nih.gov/ij/). Cells were analyzed by selecting the centre of their cell bodies, and the number of intersections at circles of increasing radii from the centre was counted using an ImageJ plugin for Sholl analysis.^34^

### General protocol for behavioural tests

Mice used in this study had been backcrossed more than nine times to the C57BL/6J strain. Behavioural tests were performed with male mice that were at least 10-weeks-old. In each strain, we ran the experiments with a single batch (*CHAMP1*^+/+^  *n* = 20, *CHAMP1*^+*/−*^  *n* = 20). Mice were group-housed in a room with a 12 h light/dark cycle (light on at 7:00 am) with access to food and water except for the period during which the olfactory test was being conducted. Room temperature was kept at 23 ± 2°C. Behavioural tests were performed between 08:00 am and 06:00 pm. After tests, all apparatus used was cleaned with diluted hypochlorite solution or 70% ethanol to prevent bias due to olfactory cues. The interval between tests was at least 1 day. Unless otherwise noted, behavioural tests were performed as previously described.^35^

### *T*-maze forced alternation test

The forced alternation test was performed using an automatic *T*-maze apparatus with a video camera (O’HARA & Co.). It was constructed of 25 cm-high white plastic runways and partitioned off into six areas by sliding doors that can be opened downward. The stem of the T was comprised area S2 (12 × 24 cm), and the arms of the T comprise areas A1 and A2 (11.4 × 20.5 cm). Areas P1 and P2 were connecting passageways from respective arms (A1 and A2) to the start compartment area (S1). Mice were subjected to a forced alternation protocol for 7 days (one session consisting of 10 trials per day; cut-off time of 50 min). Each trial had first and second runs. On the first run, the mouse was forced to choose one of the arms of area (A1 or A2). After the mouse stayed for 10 s, the door that separated the arms of area (A1 or A2) and connecting passageway (P1 or P2) was opened to allow the mouse to return to the starting area (S1). The mouse was given a 3 s delay in area S1, followed by a free choice between both T arms. The correct response was the choice of the other arm that had not been chosen by the first trial. The location of the sample arm (left or right) was varied pseudo-randomly across trials. On Days 4–7, a delay (3, 30, 60 or 120 s) was applied after trial. The percentage of correct response was measured. Data acquisition, control of sliding doors and data analysis were performed by ImageTM software (see ‘Data analysis’).

### Contextual and cued fear conditioning test

A contextual and cued fear conditioning test was conducted as previously described.^36,37^ This was performed using an automatic apparatus with a video camera (O’HARA & Co.). Each mouse was placed in an acrylic chamber (33 × 25 × 29 cm) consisting of white (side) and transparent (front, rear and top) walls with a stainless-steel grid floor (0.2 cm diametre, spaced 0.5 cm apart) (O’HARA & Co.) illuminated at 100 lux and allowed to explore freely for 2 min. White noise at 55 dB, which served as the conditioned stimulus (CS), was presented for 30 s, followed by a mild (2 s, 0.3 mA) foot shock, which served as the unconditioned stimulus (UCS). Two more CS–UCS pairings were presented with a 2 min interstimulus interval. Context test was conducted 1 day and 1 month after conditioning in the same chamber for 5 min on each mouse. Cued test with altered context was conducted after conditioning using a triangular box (31 × 31 × 32 cm) made of white opaque Plexiglas, which was located in a different room. The mice were allowed to explore the chamber for 180 s. Tone stimulus for the cued test (55 dB white noise) was applied for 3 min. In each test, freezing percentage and distance travelled (cm) were calculated automatically using ImageFZ software (see ‘Data analysis’).

### Hot plate test

The hot plate test was used to evaluate sensitivity to a painful stimulus. Mice were placed on a hot plate (Columbus Instruments) at 55.0 ± 0.1°C. The latency to the first forepaw or hind paw response defined as either a foot shake or a paw lick, was recorded with 15 s cut-off time.

### Barnes maze test

Barnes maze test was conducted as previously described.^38^ This test was performed on ‘dry land’, a white circular surface, 1.0 m in diametre, with 12 holes equally spaced around the perimetre in an automatic apparatus with a video camera (O’HARA & Co.). A black Plexiglas escape box (17 × 13 × 7 cm), which had paper cage chip on the bottom, was located under one of the holes. The hole above the escape box represented the target. The location of the target was consistent for a given mouse but was randomized across mice. The maze was rotated daily, with the spatial location of the target unchanged with respect to the distal visual room cues, to prevent a bias based on olfactory or proximal cues within the maze. During acquisition test, each trial ended when a mouse entered the escape box. If the mouse did not enter the target hole within 300 s, it was gently guided to the target hole by the experimenter. A probe trial was performed without the escape box 1 day after the last training session. The second probe trial was conducted 1 month after the last training session to evaluate memory retention. Latency, distance, and number of errors before first reaching the target hole, omission error to reach the target hole, and time spent around each hole were recorded with the use of ImageBM software (see ‘Data analysis’).

### Social interaction test in a novel environment

In this test, two mice of identical genotypes that were previously housed in different cages were placed in a box together (40 × 40 × 30 cm) (O’HARA & Co.) and allowed to explore freely for 10 min. Behaviour was recorded and analyzed automatically using ImageSI software (see ‘Data analysis’). The total number of contacts, total duration of contact, mean duration per contact and total distance travelled were measured. If the two mice contacted each other and the distance travelled by either mouse was longer than 10 cm, the behaviour was classified as an ‘active contact’. Images were captured at three frames per second and distance travelled between two successive frames was calculated for each mouse.

### Crawley’s sociability and social novelty preference test

This test is a method to investigate the effect of complex genetics on sociability and preference for social novelty.^39^ The testing apparatus consisted of a rectangular, three-chambered box and a lid with a video camera (O’HARA & Co.). Each chamber was 20 × 40 × 47 cm and the dividing walls were made from clear Plexiglas with small square openings (5 × 3 cm) allowing access into each chamber. The centre of the field was illuminated at 100 lux. A habituation session was performed in the apparatus for 10 min before the sociability test, and the wire cages in the lateral compartments were located in the corners of each compartment. In the sociability test, an unfamiliar C57BL/6J male mouse (stranger) that had no prior contact with the subject mice, was placed in one of the side chambers. The location of stranger mouse in the left or right side-chamber was systematically alternated between trials. The stranger mouse was enclosed in a small, circular wire cage, which allowed nose contact between the bars, but prevented fighting. The cage was 11 cm in height, with a bottom diametre of 9 cm, and vertical bars 0.5 cm apart. The subject mouse was first placed in the middle chamber and allowed to explore the entire test box for a 10 min session. The amount of time spent in each wire cage was measured by automatic apparatus with a video camera (O’HARA & Co.) fitted on top of the box. After the first 10 min, each mouse was tested in a second 10 min session to quantify social preference for a new stranger mouse. The new stranger mouse was enclosed in an identical small wire cage. The test mouse thus had a choice between the first, already-investigated mouse (familiar mouse) and the novel unfamiliar mouse (stranger mouse). The amount of time spent around each wire cage and chamber during the second 10 min session was recorded. Data acquisition and analysis were performed automatically using ImageCSI software (see ‘Data analysis’).

### Twenty-four-hour home-cage monitoring

Two mice of the same genotype that had been housed separately were placed together in a home cage (15 × 25 × 30 cm) (O’HARA & Co.) with video camera attached. Their social behaviours and locomotor activities were monitored in a home cage for 1 week and the values for Days 4 through 6 were averaged. The counting of mice was designated as a ‘particle’ that was detected in each frame: two particles indicated that the mice were not in contact with each other and one particle indicated contact between the two mice. Analysis was performed automatically using ImageHA software (see ‘Data analysis’).

### Rotarod test

This test, which uses an accelerating rotarod (UGO Basile), was performed on rotating drums (3 cm diametre) and latency to fall from the rotarod was recorded. The speed of the rotarod accelerated from 4 to 40 r.p.m. over a 5 min period. The test was performed three times per day.

### Grip strength test

Grip strength was measured by a grip strength metre (O’HARA & Co.). After mice grasped a wire grid by the forelimbs, they were pulled backward until they release it. The peak force applied by the forelimbs of the mouse was recorded in Newtons (N). Each mouse was tested three times, and the greatest value obtained was used for further data analysis.

### Wire-hang test

Wire-hang test apparatus was used to assess balance and grip strength. The apparatus consists of a box (21.5 × 22 × 30 cm) with a wire-mesh grid (10 × 10 cm) on top that can be inverted (O’HARA & Co.). The mice were placed on the wire mesh at the top of the apparatus, then the wire mesh was gently turned inside out. The latency to the mouse falling was recorded, with a 60 s cut-off time.

### Beam test

Beam test was conducted as previously described.^40^ Motor coordination and balance were assessed with the test. This was performed by automatic apparatus with a video camera (O’HARA & Co.). The beam test was measuring the ability of mice to traverse a narrow beam to reach a dark box. The beams, with a rough painted surface, consisted of two different strips of iron. Each beam is 100 cm long, one was 2.8 cm (wide bar) and the other was 1.1 cm (narrow bar) placed horizontally 50 cm above the surface. One session of four trials was performed using the 2.8 cm beam and the other one of five trials was performed using the 1.1 cm beam. Mice were allowed up to 90 s to traverse each beam. The number of sideslips was recorded for each trial by the ImageBT programme (see ‘Data analysis’).

### Porsolt forced swim test

Porsolt forced swim test was conducted as previously described,^37^ performed by automatic apparatus with a video camera (O’HARA & Co.). The apparatus was consisted of four Plexiglas cylinders (22 cm in height and 11 cm in diametre) that were filled with dilute sodium hypochlorite solution (∼23°C) up to a height of 7.5 cm of a white plastic chamber. The mice were placed in the cylinders, and the immobility and the distance travelled were recorded for 10 min. The test was performed for 2 days. Data acquisition and analysis were automatically performed. Immobility time (%) and distance travelled (cm) were measured by ImageTS software (see ‘Data analysis’).

### Tail suspension test

Tail suspension test was conducted as previously described.^41,42^ Mice were suspended 30 cm above the floor of a white plastic chamber (O’HARA & Co.) and recorded for 10 min. This was performed by automatic apparatus with a video camera (O’HARA & Co.). Data acquisition and analysis were automatically performed. Immobility time (%) was measured by ImageTS software (see ‘Data analysis’).

### Open field test

Each mouse was placed in the corner of the open field apparatus (40 × 40 × 30 cm) (Accuscan Instruments), which was illuminated at 100 lux. Total distance travelled (cm), vertical activity (rearing measured by counting the number of photobeam interruptions), time spent in the centre area (20 × 20 cm), and beam-break counts for stereotyped behaviours (defined by the number of breaks of the same beam) were recorded. Data were collected for 120 min.

### Light/dark transition test

The apparatus for this test was conducted in a cage (21 × 42 × 25 cm) that was divided into two sections of equal size by a partition with a door (O’HARA & Co.). One chamber was made of white plastic and brightly illuminated, whereas the other was black and dark. Mice were placed in the dark side and allowed to move freely between two chambers with the door open for 10 min. The number of transitions between the two compartments, latency to first entry into the light chamber, distance travelled and time spent in each chamber were recorded with the use of ImageLD software (see ‘Data analysis’).

### Elevated plus maze test

This test was conducted to assess anxiety-like behaviour. The elevated plus maze consisted of two open arms (25 × 5 cm) with 3 mm high transparent walls (O’HARA & Co.). The floors of the arms and the central square (5 × 5 cm) were made of white plastic plates and elevated to a height of 55 cm above the floor. The arms of the same type were arranged on opposite sides of the maze. The centre of the maze was illuminated at 100 lux. Each mouse was placed into the centre of the maze facing one of the closed arms and was recorded for 10 min. The distance travelled, number of total entries into the arms, percentage of entries into the open arms and percentage of time spent in the open arms were measured for 10 min using ImageEP software (see ‘Data analysis’).

### Startle response and prepulse inhibition test

To measure acoustic startle response and prepulse inhibition (PPI), a startle reflex measurement system (O’HARA & Co.) was used. Before this test, mice were kept in a soundproof room separate from the testing room. A test session began by placing a mouse in a transparent PVC plastic cylinder where it was left undisturbed for 10 min. White noise (40 ms) was used as the startle stimulus for all trial types. The startle response was recorded for 400 ms starting with the onset of the startle stimulus. The background noise level in each chamber was 70 dB. A test session consisted of six trial types (i.e. two types for startle stimulus-only trials, and four types for PPI trials). The intensity of the startle stimulus was 110 or 120 dB. The prepulse sound was presented 100 ms before the startle stimulus, and its intensity was 74 or 78 dB. Four combinations of prepulse and startle stimuli were used (74–110, 78–110, 74–120 and 78–120 dB). Six blocks of the six trial types were presented in a pseudo-random order, such that each trial type was presented once within a block. The average inter-trial interval was 15 s (range: 10–20 s).

### RNA sequence analysis

Total RNA was isolated from whole brain of male mouse embryos at embryonic Day 14.5 (E14.5, wild-type: *n* = 6, *CHAMP1^+/−^*; *n* = 4, *CHAMP1*^−/−^; *n* = 5) using RNeasy mini kit (QIAGEN), following the manufacturer’s instructions. Library construction and RNA-seq were performed using the Illumina sequencing platform (Annoroad Gene Technology). The short reads were mapped to the mm9 mouse reference genome using the STAR software package.^43^ The counts per million (CPM) were quantified from aligned reads by Cuffquant and Cuffnorm from the Cufflinks package and used for further analysis. The genes were annotated to the GENCODE annotation release M1 (NCBIM37), and the mitochondrial RNA and the ribosomal RNA were masked using handmade mtRNA/rRNA annotation list. Read counts per gene were obtained with HTSeq.^44^ Differentially expressed genes (DEGs) analysis and gene ontology (GO) annotation analysis were performed using iDEP.92.^45^ If none of the samples have the CPM (counts per million) values of more than 0.5 for a gene, the gene was discarded in the pre-process section in iDEP.92. The DEG was obtained under the minimum fold change >1.2 and false discovery rate (FDR) <0.1. Pre-ranked gene set enrichment analysis (GSEA) was performed using the GSEA software v.4.0.1 (Broad Institute).^46^ All (23 468) genes were ranked by fold change value in DEG analysis and used for pre-ranked GSEA. The gene set of 94 genes, in which damaging *de novo* mutations were found in NDD, were obtained from a previous report.^6^ To evaluate the significance of the enrichment of genes in the gene set in GSEA, random gene sets were created using Random Gene Set Generator (http://www.molbiotools.com/randomgenesetgenerator.html) and used for analysis together with the gene set.

### Data analysis

Length in Fig. 3C, Supplementary Fig. 1C, H, 6C and D, and signal intensity in Fig. 4D were measured using Fiji.^47^ Angles in Fig. 3E were measured using MATLAB R2017b (Mathworks). The applications for behavioural studies (ImageTM, ImageFZ, ImageBM, ImageCSI, ImageHA, ImageBT, ImageTS, ImageLD, and ImageEP) were developed by Tsuyoshi Miyakawa (available through O’HARA & Co.) and are based on ImageJ. Statistical analysis according to paired *t*-test, Student’s *t*-test, one-way ANOVA, or two-way repeated-measures ANOVA was performed with the use of StatView 5.0.1 software (SAS Institute). Statistical analyses using the Tukey–Kramer *post hoc* test and the Steel–Dwass *post hoc* test were performed with Easy R (EZR),^48^ which is a graphical user interface for R [R Core Team, R: a language and environment for statistical computing, https://www.R-project.org/, (2018)]. More precisely, it is a modified version of R commander designed to add statistical functions frequently used in biostatistics. Data are represented as mean ± standard error of the mean (S.E.M.). If a *P* value was smaller than 0.05, the difference was considered statistically significant.

### Data availability

The data that support the finding of this study are available from the corresponding author, upon reasonable request.

## Results

### *CHAMP1* ^−/−^ mice were lethal at newborn

To explore the function of CHAMP1 *in vivo*, we generated a *CHAMP1* knockout mouse. We first generated a transgenic mouse in which the *CHAMP1* genomic region containing exon 2 was replaced with a gene cassette containing a neomycin resistant gene and lox-P sequences (Fig. 1B). We verified homologous recombination by Southern blot analysis (Fig. 1C). The *CHAMP1* knockout mice were generated by excising the gene cassette by crossing with mice systemically expressing Cre recombinase under CAG promoter (CAG-Cre; Fig. 1B), which was confirmed by a polymerase chain reaction analysis (Fig. 1D), an IB analysis (Fig. 1E), as well as an immunofluorescence analysis of MEFs (Supplementary Fig. 1A). On C57BL/6J background, *CHAMP1^+/−^* mice were healthy and fertile, and *CHAMP1*^−/−^ embryos were produced from heterozygous intercrosses at approximately the frequency predicted by Mendel’s law (Fig. 1F). However, *CHAMP1*^−/−^ pups died within 2 days of birth (Fig. 1F). The newborn *CHAMP1*^−/−^ mice looked superficially normal (Fig. 1G), although slightly smaller compared with wild-type mice (Fig. 1H). Absolute brain weight and ratios of brain weight to body weight showed no significant difference between wild-type and *CHAMP1* knockout mice (Fig. 1I–K). Measurements using various parameters of cortical size, such as anteroposterior length, cortical length, and cortical width, also showed no difference between wild-type and *CHAMP1* knockout mice (Supplementary Fig. 1B and C). We also measured these parameters in 13-week-old adult mice and found that there were no significant differences between wild-type and *CHAMP1^+/−^* mice (Supplementary Fig. 1D-H).

To explore the neonatal lethal phenotype of *CHAMP1*^−/−^ mice further, we crossed *CHAMP1^+/−^* mice on C57BL/6 background with wild-type mice on Balb/c background and obtained *CHAMP1^+/−^* mice on mixed genetic background (Supplementary Fig. 2A). Intriguingly, when we crossed these *CHAMP1^+/−^* mice with mixed backgrounds, we could obtain *CHAMP1*^−/−^ mice that survived to adulthood, although the frequency was lower than that predicted by Mendel’s law (Supplementary Fig. 2B). The *CHAMP1*^−/−^ mice on mixed background were significantly smaller than their *CHAMP1^+/+^* or *CHAMP1^+/−^* littermates (Supplementary Fig. 2C and D), and some of them exhibited kinked tails and kyphosis (Supplementary Fig. 2C). Expression of CHAMP1 and POGZ in adult brains was comparable between C57BL/6 and Balb/c backgrounds (Supplementary Fig. 2E). When we crossed the *CHAMP1^+/−^* mice on mixed background further with wild-type mice on Balb/c background to increase the contribution of Balb/c genetic background, the number of *CHAMP1*^−/−^ mice obtained by crosses between these *CHAMP1^+/−^* mice decreased (Supplementary Fig. 2B), suggesting that *CHAMP1*^−/−^ mice can survive to adulthood on mixed background, but not on pure genetic background. Systemic analysis of structural abnormalities of various organs in *CHAMP1*^−/−^ mice using sagittal sections of newborn mice on C57BL/6 or mixed backgrounds did not reveal significant differences compared to *CHAMP1^+/+^* or *CHAMP1^+/−^* mice (data not shown), leaving the cause of the neonatal death of *CHAMP1*^−/−^ mice unspecified.

### CHAMP1 localizes throughout the brain

**Figure 2:**
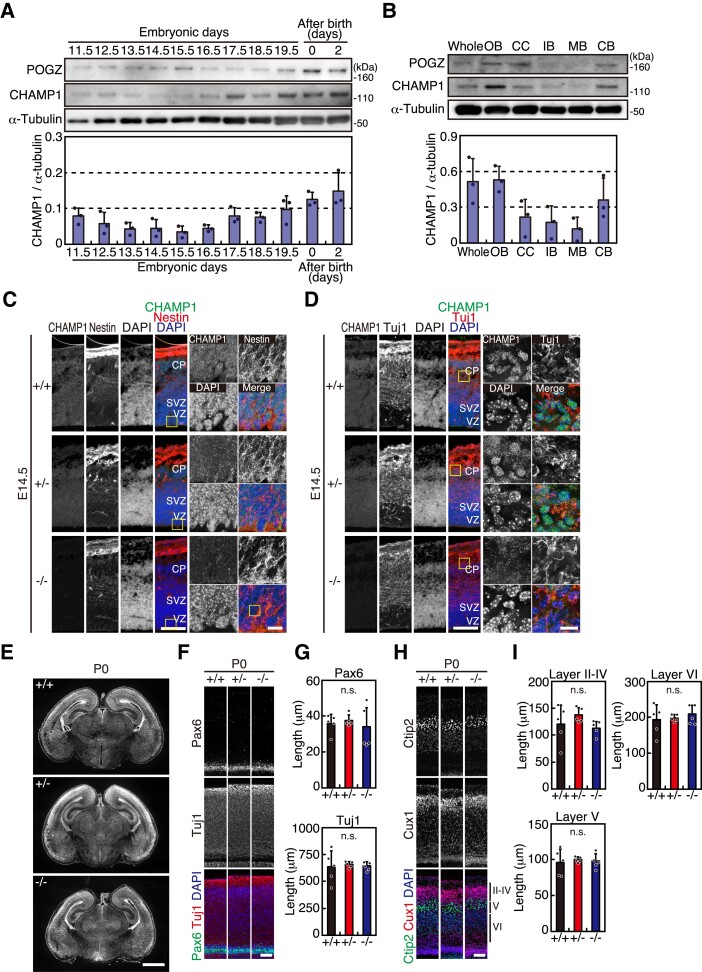
***CHAMP1* ^−/−^ mice do not show gross defects in the brain structure.** (**A**) Expression of CHAMP1 and POGZ in brain during development. Whole-cell lysates prepared from mouse brains at indicated periods were subjected to IB analysis using antibodies as indicated. Quantification results of CHAMP1 are shown at the bottom (*n* = 3). Error bars represent S.D. (**B**) Expression of CHAMP1 and POGZ in whole brain, olfactory bulb (OB), cerebral cortex (CC), interbrain (IB), midbrain (MB), and cerebellum (CB) from newborn mice. Whole-cell lysate prepared from each part of the brain was subjected to IB analysis using antibodies as indicated. Quantification results of CHAMP1 are shown at the bottom (*n* = 3). Error bars represent S.D. (**C, D**) Immunofluorescence staining of a large dorsal cortical region of E14.5 *CHAMP1*^+/+^ (upper), *CHAMP1*^+*/−*^ (middle), and *CHAMP1*^−/−^ (lower) mice using antibodies against CHAMP1 (green) and Nestin (**C**) or Tuji1 (**D**) (red). Nuclei were stained with DAPI (blue). Scale bars: 100 μm. Scale bars in close-up images of boxed area: 10 μm. CP, cortical plate; SVZ, sub-ventricular zone; VZ, ventricular zone. (**E**) Nissl staining of coronal sections of newborn *CHAMP1*^+/+^ (upper), *CHAMP1*^+*/−*^ (middle), and *CHAMP1*^−/−^ (lower) mice. Scale bar: 1 mm. (**F**) Immunofluorescence staining of a large dorsal cortical region of newborn *CHAMP1*^+/+^, *CHAMP1*^+*/−*^, and *CHAMP1*^−/−^ mice using antibodies against Pax6 (green) and Tuj1 (red). Nuclei were stained with DAPI (blue). Scale bar: 50 μm. (**G**) Thickness of the Pax6 (upper) and Tuj1 (lower)-positive cell layer of newborn mice shown in (**F**) (*CHAMP1*^+/+^; *n* = 5, *CHAMP1*^+*/−*^; *n* = 5, *CHAMP1*^−/−^; *n* = 5). Error bars represent S.D. *P* values were determined by Tukey–Kramer test. n.s., not statistically significant. (**H**) Immunofluorescence staining of a large dorsal cortical region of newborn *CHAMP1*^+/+^, *CHAMP1*^+*/−*^, and *CHAMP1*^−/−^ mice using antibodies against Ctip2 (green) and Cux1 (red). Nuclei were stained with DAPI (blue). Scale bar: 50 μm. II〜VI, cortical layers. (**I**) Thickness of the Ctip2 (II–IV) and Cux1 (V)-positive cell layers of newborn mice shown in (**H**) (*CHAMP1*^+/+^; *n* = 5, *CHAMP1*^+*/−*^; *n* = 5, *CHAMP1*^−/−^; *n* = 5). Thickness of the layer VI was also measured. Error bars represent S.D. *P* values were determined by Tukey–Kramer test. n.s., not statistically significant.

We observed CHAMP1 expression in the brain. In an IB analysis, CHAMP1 expression increased at E17.5 and thereafter, i.e. the stages when neuronal production is almost completed (Fig. 2A), and it is expressed in each part of the neonatal brain in varying degrees (Fig. 2B). Expression of POGZ, a binding partner of CHAMP1, increased at E14.5-E15.5 and after birth (Fig. 2A, Supplementary Fig. 3A). POGZ showed a similar expression pattern in the neonatal brain as CHAMP1 (Fig. 2B, Supplementary Fig. 3B). By immunostaining of brain sections of E12.5, E14.5, neonatal, and adult mice, CHAMP1 was detected in each part of the brain, localizing in the nucleus of individual cells (Supplementary Fig. 3C-F). CHAMP1 was not detected in the brain sections of *CHAMP1*^−/−^ mice, confirming the specificity of the fluorescent signal. *In situ* hybridization using brain sections obtained from E14.5 mice showed the expression of *CHAMP1* mRNA throughout the brain, which was not detected in *CHAMP1*^−/−^ mice (Supplementary Fig. 3G). We also examined *POGZ* mRNA in E14.5 mouse brains and found that its distribution is not altered in *CHAMP1* knockout mice (Supplementary Fig. 4A). In E14.5, neonatal, and adult brains, POGZ localizes in the nucleus of the cells throughout the brain (Supplementary Fig. 4B–D), consistent with the previous reports.^25,49,50^ The nuclear localization of POGZ was detected in *CHAMP1* knockout mice (Supplementary Fig. 4B–D), suggesting that CHAMP1 is not essential for POGZ localization in the nucleus. In immunostaining of cortical sections of E14.5 mice, CHAMP1 was detected in both NSCs and neurons, which were stained with Nestin and Tuj1, respectively (Fig. 2C and D). Overall, CHAMP1 localizes throughout the brain in both embryonic and adult mice, and the expression was increased during the embryonic period.

### *CHAMP1* knockout mice do not show gross defects in brain structure

We morphologically and histologically analyzed the brain of neonatal *CHAMP1* knockout mice. In Nissl staining of brain sections, no gross anatomical abnormalities were found between wild-type and *CHAMP1* knockout mice (Fig. 2E). When we examined NSCs and neurons in the dorsal cortical area by Pax6 and Tuj1 immunofluorescence, respectively, no significant difference in the thickness of the Pax6 and Tuj1-positive cell layer were found between wild-type and *CHAMP1* knockout newborn mice (Fig. 2F, G). The difference was also not apparent in E14.5 mice (Supplementary Fig. 5A). We also compared the thickness of the cortical layers. There was no significant difference in the thickness of the Ctip2 and Cux1-positive cell layers that represent layer V and layer II–IV, respectively (Fig. 2H and I), in newborn and adult brain of *CHAMP1* knockout mice (Fig. 2H and I, Supplementary Fig. 5B). Collectively, the brain structure of *CHAMP1* knockout mice appeared grossly normal.

### Mitotic cells are increased in *CHAMP1* knockout mouse brains

**Figure 3:**
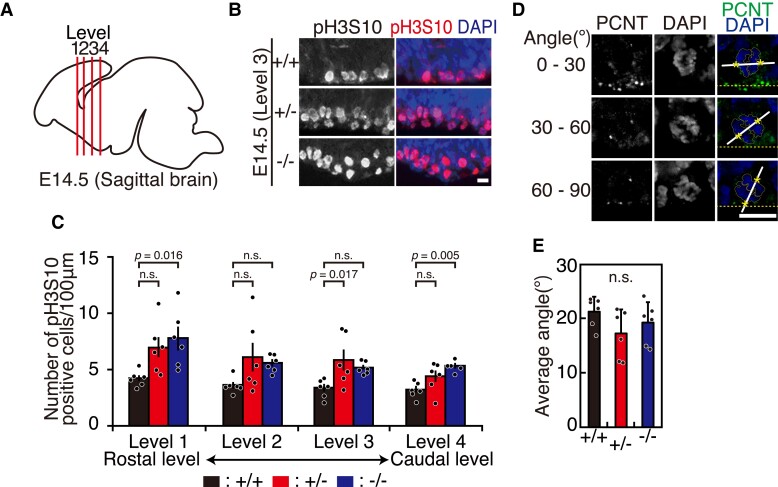
**Mitotic cells are increased in *CHAMP1* knockout mouse brains.** (**A**) A schematic diagram of E14.5 brain depicting the levels of coronal sections, in which phospho-histone H3 at serine 10 was visualized. (**B**) Immunofluorescence staining of the VZ of E14.5 *CHAMP1*^+/+^ (upper), *CHAMP1*^+^*^/−^* (middle), and *CHAMP1*^−/−^ (lower) mice at Level 3 in (**A**), using antibodies against phosphorylated histone H3 at serine 10 (pH3S10, red). Nuclei were stained with DAPI (blue). Scale bar: 10 μm. (**C**) Quantitative analysis of the number of pH3S10-positive cells in VZ of E14.5 mice at different levels shown in (**A**) (*CHAMP1*^+/+^; *n* = 6 (cortices), *CHAMP1*^+^*^/−^*; *n* = 6, *CHAMP1*^−/−^; *n* = 6). The data represent a minimum of 18 cells per cortex. Error bars represent S.E.M. *P* values were determined by Tukey–Kramer test. n.s., not statistically significant. (**D**) Immunofluorescence images of anaphase progenitor cells of E14.5 mice stained with antibodies against PCNT (green). Representative cells showing different orientation of the anaphase spindle axis to the ventricular surface are shown. Nuclei were stained with DAPI (blue). Centrosome positions, determined by PCNT signals, were shown by asterisks. Scale bar: 10 μm. (**E**) Average of the angle between anaphase spindle axis and the ventricular surface in *CHAMP1*^+/+^ (six cortices from three mice, at least 20 cells were counted per cortex), *CHAMP1*^+^*^/−^* (six cortices from three mice, at least 18 cells were counted per cortex), and *CHAMP1*^−/−^ (six cortices from three mice, at least 16 cells were counted per cortex) mice. Error bars represent S.D. n.s. not statistically significant (Tukey–Kramer test).

Since CHAMP1 is involved in chromosome segregation,^11^ we looked for mitotic defects in the brain of *CHAMP1* knockout mice. We compared the number of mitotic progenitor cells in *CHAMP1* knockout mice with that of wild-type mice by visualizing phospho-histone H3 at serine 10 (pH3S10), a mitotic phase marker, in E14.5 brains. To adjust the relative position along the longitudinal axis, we compared the number of cells positive for pH3S10 at four different levels along the longitudinal axis (Level 1–4, Fig. 3A). As shown in Fig. 3B and C, the number of pH3S10-positive cells tended to increase at the VZ in *CHAMP1* knockout mice. Considering the role of CHAMP1 in chromosome segregation, the increase of mitotic cells may represent delay in mitotic progression rather than accelerated cell cycle. We did not find increase in apoptotic cells in the brain in E14.5 or neonatal *CHAMP1* knockout mice compared with corresponding wild-type mice by terminal deoxynucleotidyl TUNEL staining (Supplementary Fig. 6A), suggesting that the mitotic delay is not severe enough to cause increased cell death. We further examined the orientation of the spindle axis in mitotic progenitor cells relative to the ventricular surface, because this has been proposed to influence cell fate determination.^51^ However, there was no significant difference between wild-type and *CHAMP1* knockout E14.5 brains when we measured the orientation of the spindle axis (Fig. 3D and E).

Disruption of dendritic spines, membrane protrusions along dendrites, is found in patients with NDDs.^52^ We visualized and quantified the density of dendritic spines in the cerebral cortex and hippocampus of 13 week-old mice using Golgi-Cox staining, but found no difference between wild-type and *CHAMP1^+/−^* mice (Supplementary Fig. 6B and C). The density of mature spines, which exhibit mushroom-like morphologies, was also comparable between wild-type and *CHAMP1^+/−^* mice (Supplementary Fig. 6D).

### *CHAMP1* is involved in NSC differentiation *in vitro*

**Figure 4:**
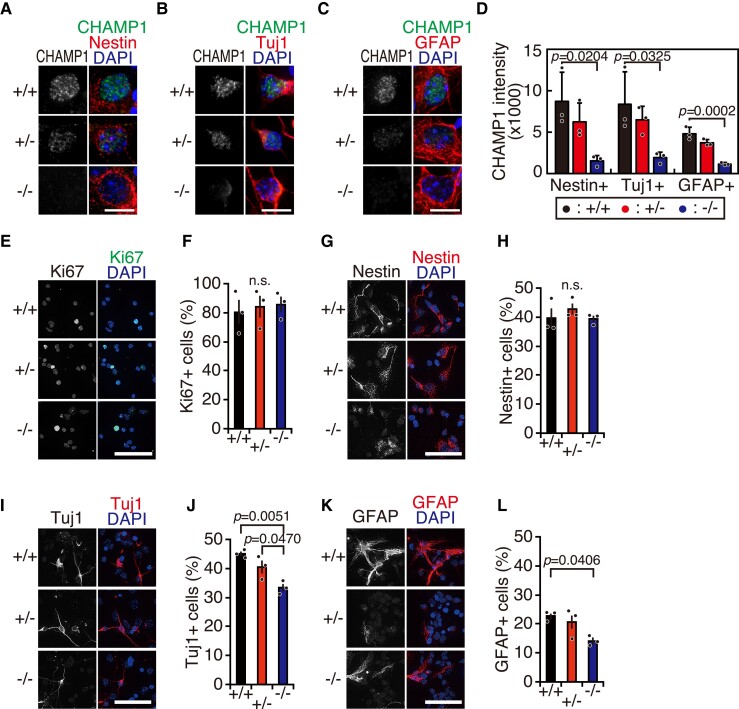
***CHAMP1* is involved in NSC differentiation and axon outgrowth.** (**A**) Immunofluorescence staining of NSCs isolated from the cortex of E14.5 *CHAMP1*^+/+^ (upper), *CHAMP1*^+*/−*^ (middle), and *CHAMP1*^−/−^ (lower) mice using antibodies against CHAMP1 (green) and Nestin (red). Nuclei were stained with DAPI (blue). Scale bar: 10 μm. (**B**) Immunofluorescence staining of neurons differentiated from NSCs of E14.5 *CHAMP1*^+/+^ (upper), *CHAMP1*^+*/−*^ (middle), and *CHAMP1*^−/−^ (lower) mice *in vitro* using antibodies against CHAMP1 (green) and Tuj1 (red). Nuclei were stained with DAPI (blue). Scale bar: 10 μm. (**C**) Immunofluorescence staining of astrocytes differentiated from NSCs of E14.5 *CHAMP1*^+/+^ (upper), *CHAMP1*^+*/−*^ (middle), and *CHAMP1*^−/−^ (lower) mice *in vitro* using antibodies against CHAMP1 (green) and GFAP (red). Nuclei were stained with DAPI (blue). Scale bar: 10 μm. (**D**) Quantification of CHAMP1 signal intensity in cultured NSCs (Nestin+), neurons (Tuj1+), and astrocytes (GFAP) isolated from E14.5 *CHAMP1*^+/+^ (*n* = 3), *CHAMP1*^+*/−*^ (*n* = 3), and *CHAMP1*^−/−^ (*n* = 3) mice. The data represent a minimum of 40 cells per mouse. Error bars represent S.D. *P* values were determined by Tukey–Kramer test. (**E**) Immunofluorescence staining of NSCs isolated from the cortex of E14.5 *CHAMP1*^+/+^ (upper), *CHAMP1*^+*/−*^ (middle), and *CHAMP1*^−/−^ (lower) mice using antibodies against Ki67 (green). Nuclei were stained with DAPI (blue). Scale bar: 50 μm. (**F**) Quantification of Ki67-positive NSCs (*CHAMP1*^+/+^; *n* = 3, *CHAMP1*^+*/−*^; *n* = 3, *CHAMP1*^−/−^; *n* = 3). The data represent a minimum of 1000 cells per mouse. Error bars represent S.E.M. *P* values were determined by Tukey–Kramer test. n.s., not statistically significant. (**G, I, K**) Immunofluorescence staining of NSCs isolated from the cortex of E14.5 *CHAMP1*^+/+^ (upper), *CHAMP1*^+*/−*^ (middle), and *CHAMP1*^−/−^ (lower) mice differentiated *in vitro* by growth factor withdrawal using antibodies against Nestin (**G**), Tuj1 (**I**), or GFAP (**K**) (red). Nuclei were stained with DAPI (blue). Scale bar: 50 μm. (**H, J, L**) Quantification of differentiated NSCs positive for Nestin (**H**), Tuj1 (**J**), or GFAP (**L**) (*CHAMP1*^+/+^; *n* = 3, *CHAMP1*^+*/−*^; *n* = 3, *CHAMP1*^−/−^; *n* = 3). The data represent a minimum of 1004 cells per mouse. Error bars represent S.E.M. *P* values were determined by Tukey–Kramer test. n.s., not statistically significant.

We enriched NSCs from the cortex of E14.5 mice by *in vitro* culture for 4 days in the presence of EGF and FGF2, which were then differentiated by growth factor withdrawal for 5 days. First, we addressed CHAMP1 expression in NSCs, neurons, and astrocytes, by immunofluorescence analysis with lineage-specific markers, Nestin, Tuj1 and GFAP, respectively. CHAMP1 was detected in cells expressing each of the respective markers, showing that CHAMP1 is expressed in NSCs, neurons and astrocytes, although expression is lower in astrocytes than in NSCs and neurons (Fig. 4A–D). The role of CHAMP1 in NSC proliferation was examined by measuring the rate of appearance of Ki67-positive cells in the presence of growth factors, and we found no difference between wild-type and *CHAMP1* knockout mice (Fig. 4E and F). We also determined the role of CHAMP1 on NSC differentiation after growth factor withdrawal by quantifying the fraction of cells expressing lineage-specific markers. The percentages of Tuj1- and GFAP-positive cells were significantly reduced in *CHAMP1*^−/−^ cells, whereas that of Nestin-positive cells was unaffected, compared with wild-type cells (Fig. 4G–L). These data suggest that CHAMP1 is involved in NSC differentiation *in vitro* to neuronal and astrocyte lineages.

We also performed CHAMP1 knockdown in NSCs from E14.5 mice and observed after differentiation for 5 days. In an IB analysis and immunofluorescence staining, we found that POGZ expression is reduced in CHAMP1 knockdown neurons (Supplementary Fig. 7A and B), suggesting that CHAMP1 is required for the stability of POGZ, which was also shown in a recent report.^53^ We also studied axon outgrowth in CHAMP1 knockdown neurons by measuring the number of axons stained with ankyrin-G, a marker of the axon initial segment, which were also positive for Map2, a dendritic marker. As shown in Supplementary Fig. 7D and E, the number of axons in CHAMP1 knockdown neurons was comparable with that in control neurons. Sholl analysis showed that the level of dendritic branching is not significantly different between CHAMP1 knockdown and control neurons (Supplementary Fig. 7F and G).

### CHAMP1 knockdown leads to delayed cortical migration

**Figure 5:**
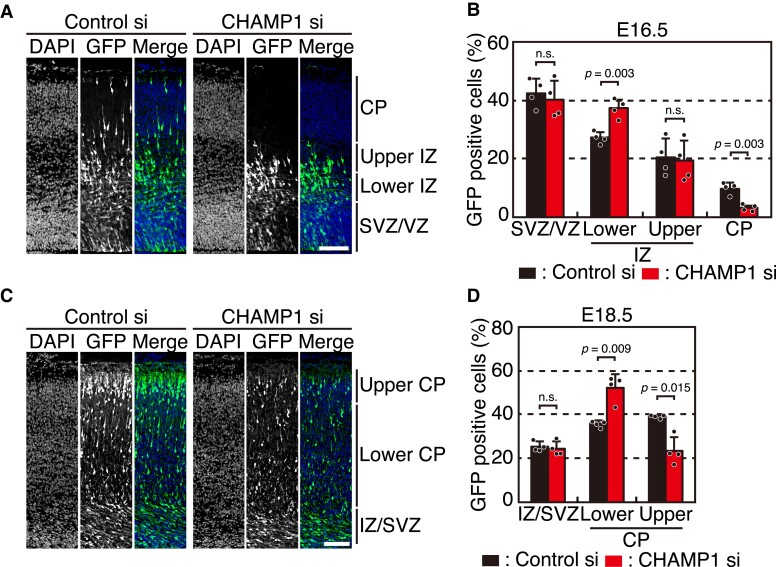
**CHAMP1 knockdown leads to delayed cortical migration.** (**A**) Cell localization in E16.5 mouse cortices electroporated with mock or *CHAMP1* siRNA at E14.5. Electroporated cells expressing GFP were shown in green. Nuclei were stained with DAPI (blue). Scale bar: 50 μm. CP, cortical plate; IZ, intermediate zone; SVZ, sub-ventricular zone; VZ, ventricular zone. (**B**) Quantification of GFP-positive cells treated with control or *CHAMP1* siRNA in each layer shown in (**A**) (*n* = 4). Error bars represent S.D. *P* values were determined by Student’s *t*-test. n.s., not statistically significant. (**C**) Neuronal migration in E18.5 mouse cortices electroporated with mock or *CHAMP1* siRNA at E14.5. Electroporated cells expressing GFP were shown in green. Nuclei were stained with DAPI (blue). Scale bar: 50 μm. (**D**) Quantification of GFP-positive cells treated with control or *CHAMP1* siRNA in each layer shown in (**C**) (*n* = 4). Error bars represent S.D. *P* values were determined by Student’s *t*-test. n.s., not statistically significant.

To determine the role of CHAMP1 in NSCs of the developing cortex *in vivo*, we introduced an siRNA against *CHAMP1* and an expression plasmid for GFP into the E14.5 mouse cortex by *in utero* electroporation. The embryos were analyzed for the localization of GFP-expressing cells in the developing cortex at E16.5 and E18.5. Some of the GFP-positive cells reached the cortical plate (CP) in the control embryos at E16.5, whereas most of the cells transfected with the *CHAMP1* siRNA were localized in the VZ, sub-ventricular zone (SVZ) and intermediate zone (IZ) but not in the CP (Fig. 5A). The quantitative data showed that the fraction of GFP-positive cells was significantly increased in the lower IZ yet decreased in the CP in CHAMP1 knockdown embryos (Fig. 5B). Considering the results *in vitro* (Fig. 4G–L), these data suggest that CHAMP1 might be involved in promoting neuronal differentiation. At E18.5, the fraction of GFP-positive cells was increased in the lower CP and decreased in the upper CP (Fig. 5C and D), implicating that NSCs with CHAMP1 knockdown might be delayed in neuronal differentiation or have a defect in neuronal migration. The reason why the impairment of cortical cell localization was not detected in newborn mice (Fig. 2F and G) may be because the delay was small and only transiently detectable by acute CHAMP1 depletion by RNAi.

### *CHAMP1^+/−^* mice have mild memory defects

**Figure 6:**
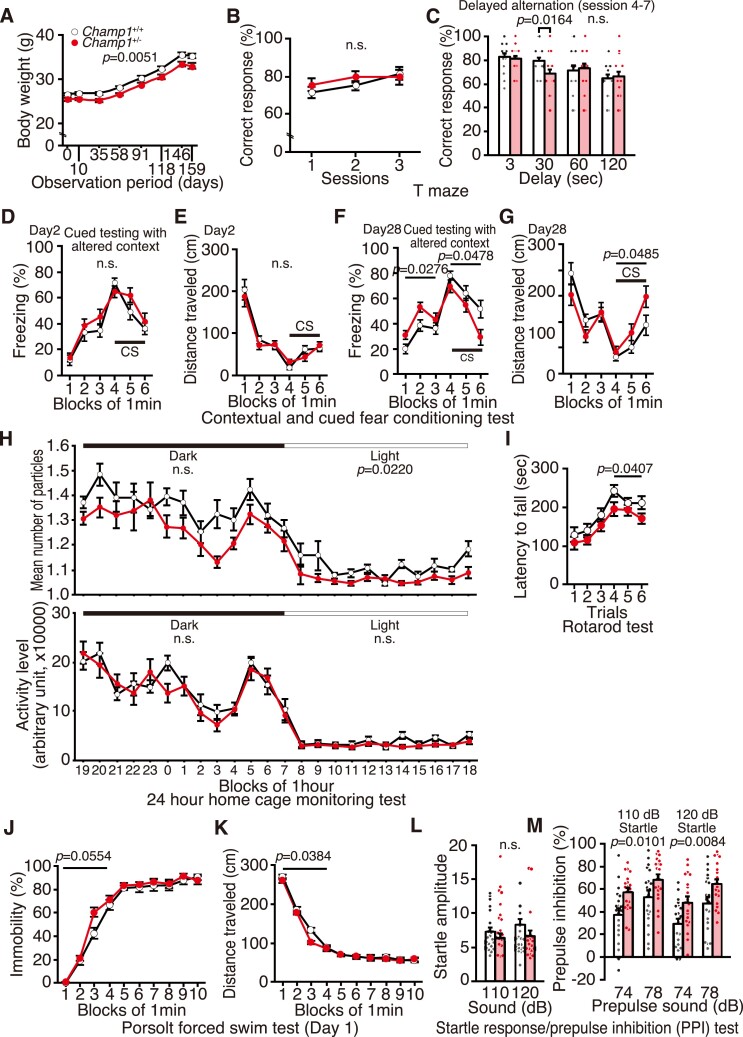
***CHAMP1^+^^/−^* mice show mild memory defects, altered social interaction and depressive behaviours.** (**A**) Body weight of *CHAMP1*^+/+^ (white circle; *n* = 20) and *CHAMP1*^+*/−*^ (red circle; *n* = 20) mice during 10–37 weeks-old, plotted against observation period of the behavioural test battery. (**B, C**) *T*-maze forced alteration test. Percentage of correct response in the first three training (**B**) and the four consecutive sessions with various delay times (**C**) of *CHAMP1*^+/+^ (white bars) and *CHAMP1*^+*/−*^ (red bars) mice. (**D-G**) Cued and contextual fear conditioning test: (**D**) percentage of freezing time and (**E**) total distance moved in the chamber on Day 2, and (**F**) percentage of freezing time and (**G**) total distance moved in the chamber after 1 month of *CHAMP1*^+/+^ (white circle) and *CHAMP1*^+*/−*^ (red circle) mice. CS: conditioned stimulus. (**H**) Social interaction test in home cage: mean number of particles, which represents the contact between two mice (upper graph, one particle: two mice were together, two particles: they were separating), and mean activities (lower graph) of *CHAMP1*^+/+^ (white circle) and *CHAMP1*^+*/−*^ (red circle) mice. (**I**) Latency to fall of *CHAMP1*^+/+^ (white circle) and *CHAMP1*^+*/−*^ (red circle) mice in the rotarod test. (**J, K**) Porsolt forced swim test: (**J**) percentage of immobility and (**K**) total distance moved on Day 1 by *CHAMP1*^+/+^ (white circle) and *CHAMP1*^+*/−*^ (red circle) mice. (**L, M**) Startle response and PPI test. Acoustic startle response to stimuli of 110 or 120 dB (**L**), and inhibition of the startle response by prepulse of 74 or 78 dB (**M**) in *CHAMP1*^+/+^ (white bars) and *CHAMP1*^+*/−*^ (red bars) mice. For all graphs, twenty male mice of both genotypes were used, and error bars represent S.E.M. *P* values for genotype effects were determined by two-way repeated measurement ANOVA. In (**C**) at 30 s, Student’s *t*-test was used. In (**F**) and (**G**), data before CS (1–3 min) and during CS (4–6 min) were analyzed separately. In (**I**), data for the 1st to 3rd trials and the 4th to 6th trials were analyzed separately. In (**L**) and (**M**), data for early time points (1–4 min) and late time points (5–10 min) were analyzed separately.

The results so far show that neuronal defects in *CHAMP1^+/−^* mice are trending in the same direction, although not as strong, as that in *CHAMP1*^−/−^ mice. Next, we performed a comprehensive battery of behavioural tests, which is available only for *CHAMP1^+/−^* mice, because *CHAMP1*^−/−^ mice die soon after birth. We used 20 adult males of both wild-type and *CHAMP1^+/−^* mice at 10-weeks-old at test onset, which were backcrossed at least nine generations from the original C57BL/6N × CBA/J mix genetic background to generate C57BL/6J genetic background. *CHAMP1^+/−^* mice used for the behavioural test battery showed a small but significant decrease in body weight throughout the observation period (Fig. 6A, Supplementary Fig. 8A), which was not apparent in mice that were not used for the behavioural test (Supplementary Fig. 1D). The small difference in body weight may become apparent only in the strictly controlled rearing conditions.

To evaluate working memory, a *T*-maze test was performed according to the forced alteration protocol. The percentage of correct responses of *CHAMP1^+/−^* mice was not significantly different from that of wild-type mice (Fig. 6B and C). However, there was a difference only in 30 s delay (*P* = 0.0164, Fig. 6C), which may reflect a slight impairment in working memory in *CHAMP1^+/−^* mice.

Next we performed a cued and contextual fear conditioning test to assess the involvement of CHAMP1 in fear memory. In the conditioning period, there was no significant difference between wild-type and *CHAMP1^+/−^* mice in the percentage of freezing or distance travelled caused by the foot shock delivered as the UCS following the white noise as the conditioned stimulus (CS; Supplementary Fig. 8B) but the distance travelled during the 1st electrical foot shock was greater in *CHAMP1^+/−^* mice (Supplementary Fig. 8E, foot shock 1, *P* = 0.0278). In a hot plate test, latency to the first hind paw response did not differ between wild-type and *CHAMP1^+/−^* mice (Supplementary Fig. 8F), excluding the possibility that *CHAMP1^+/−^* mice have defective sensitivity to a painful stimulus. During Day 2 of context testing, there was no difference between wild-type and *CHAMP1^+/−^* mice in the percentages of freezing or distance travelled (Supplementary Fig. 8C). In cued testing with altered context, in which CS was presented without UCS, the percentages of freezing and distance travelled were comparable between wild-type and *CHAMP1^+/−^* mice (Fig. 6D and E). In the context testing 1 month after the fear conditioning, there was no difference between wild-type and *CHAMP1^+/−^* mice in the percentages of freezing and distance travelled (Supplementary Fig. 8D). However, in the cued testing after 1 month, the percentage of freezing of *CHAMP1^+/−^* mice was significantly increased before CS (*P* = 0.0276) and decreased during CS (*P* = 0.0478, Fig. 6F). Accordingly, total distance travelled by *CHAMP1^+/−^* mice were longer during CS (*P* = 0.0485, Fig. 6G). Decreased freezing in response to CS despite the increase in generalized freezing before CS only after a month might be explained by a problem in remote, but not recent, cued memory in *CHAMP1^+/−^* mice.

We then used the Barnes maze test to measure spatial learning and memory. There were no significant differences in latency, number of errors, distance travelled and number of omission errors before reaching the target hole between wild-type and *CHAMP1^+/−^* mice (Supplementary Fig. 9A). In probe trials, in which the escape box was removed, performed either 1 day or 1 month after training, there was no significant difference between wild-type and *CHAMP1^+/−^* mice in the time spent around the target (Supplementary Fig. 9B), showing that spatial memory is not affected. Collectively, these results suggest that *CHAMP1^+/−^* mice have a cued memory defect but not a spatial memory defect.

### *CHAMP1^+/−^* mice show altered social interaction

To evaluate social interaction, a 24 h home-cage monitoring test was conducted. As shown in Fig. 6H, where mean number of particles represents the contact between two mice (one particle: two mice were together, two particles: they were separating), *CHAMP1^+/−^* mice exhibited increased interaction compared with wild-type mice, which was statistically significant in inactive periods (‘light’, *P* = 0.0220, Fig. 6H). Activity level was comparable between *CHAMP1^+/−^* and wild-type mice. These data suggest that *CHAMP1^+/−^* mice show altered social interaction.

In the social interaction test, which measures social behaviour in a novel environment, there was no difference between wild-type and *CHAMP1^+/−^* mice in all the parameters (Supplementary Fig. 9C). There was also no difference between wild-type and *CHAMP1^+/−^* mice in a Crawley’s sociability and social novelty preference test (Supplementary Fig. 9D and E), suggesting that social recognition is not impaired.

### *CHAMP1^+/−^* mice present depression-like behaviours

In a rotarod test that assesses motor coordination, latency to fall in the 4th to 6th trials were significantly shorter in *CHAMP1^+/−^* mice compared with that in wild-type mice (*P* = 0.0407, Fig. 6I). Neuromuscular strength did not differ between wild-type and *CHAMP1^+/−^* mice, which was shown by a grip strength test and a wire-hung test (Supplementary Fig. 10A and B). The finding that body weight was slightly smaller in adult *CHAMP1^+/−^* mice (Fig. 6A) also makes it unlikely that the result of the rotarod test was due to physical problems. Motor function was also assessed in a beam test. Number of slips, moving speed, and number of move episodes were no different between wild-type and *CHAMP1^+/−^* mice (Supplementary Fig. 10C). These results suggest that *CHAMP1^+/−^* mice have a tendency to give up easily through psychological but not physical reason.

Next, we examined depression-like behaviour in a Porsolt forced swim test performed on two consecutive days. Total immobility time tended to be longer in *CHAMP1^+/−^* mice only in early time points in the first day trial (*P* = 0.0554, Fig. 6J). Accordingly, total distance travelled was significantly shorter in *CHAMP1^+/−^* mice only in early time points in the first day trial (*P* = 0.0380, Fig. 6K). Total immobility time and total distance travelled did not differ between wild-type and *CHAMP1^+/−^* mice in the second day trial (Supplementary Fig. 10D). In a tail suspension test, which is also for detecting depression-like behaviour, total immobility time was not different between wild-type and *CHAMP1^+/−^* mice (Supplementary Fig. 10E). Collectively, these results imply that *CHAMP1^+/−^* mice have a minor tendency to present depression-like behaviour.

To evaluate anxiety, an open field test, a light/dark transition test, and an elevated plus maze test were performed but none showed any statistical difference between wild-type and *CHAMP1^+/−^* mice (Supplementary Fig. 10F–H). When we examined acoustic startle response and PPI, the acoustic startle response was not different between wild-type and *CHAMP1^+/−^* mice (Fig. 6L). However, *CHAMP1^+/−^* mice exhibited increased PPI for 74 dB or 78 dB prepulse before 110 dB or 120 dB startle stimulus (*P* = 0.0101, 0.0084, Fig. 6M, see Discussion).

### Gene expression changes in *CHAMP1* knockout mice

**Figure 7:**
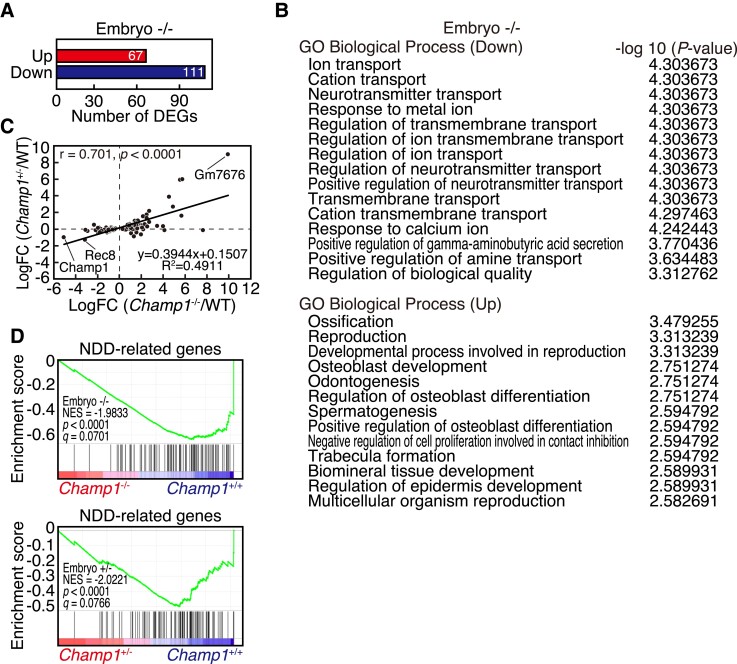
**Gene expression changes in *CHAMP1* knockout mice.** (**A**) Number of DEGs in embryonic brain of *CHAMP1*^−/−^ mice. Genes with FDR <0.1 and fold change (FC) >1.2 were selected as DEGs. (**B**) GO of biological processes enriched in DEGs in embryonic brain of *CHAMP1*^−/−^ mice. (**C**) Correlation between fold changes of gene expression in embryonic brain of *CHAMP1*^−/−^ and *CHAMP1^+/−^* mice for 178 DEGs of *CHAMP1*^−/−^ embryonic brain. Spearman’s rank correlation coefficients (*r*) and *P* values are shown. (**D**) GSEA plots of DEGs between wild-type and *CHAMP1*^−/−^ (upper) or *CHAMP1^+/−^* (lower) embryonic brain for the list of NDD-related genes. Nominal *P* values and FDR (*q*) are shown.

To explore the alteration of gene expression profiles in *CHAMP1* knockout mice, we performed RNA-seq analysis of whole brains of wild-type, *CHAMP1^+/−^*, and *CHAMP1*^−/−^ mouse embryos at E14.5. We found 178 DEGs in embryonic brain of *CHAMP1*^−/−^ mice compared with wild-type mice at cut-offs corresponding to false discovery rate (FDR) <0.1 and fold change (FC) >1.2, among which 111 were downregulated and 67 were upregulated (Fig. 7A, Supplementary Fig. 11A). A GO enrichment analysis of DEGs downregulated in *CHAMP1*^−/−^ embryonic brain showed enrichment for genes related to neurotransmitter transport (Fig. 7B), implicating the role of CHAMP1 in brain function. Among the DEGs related to neurotransmitter transport, *Slc6a1*, a gamma-aminobutyric acid (GABA) transporter associated with ID,^54^ was included (Supplementary Fig. 11A). For DEGs upregulated in *CHAMP1*^−/−^ embryonic brain, genes involved in regulation of ossification were enriched, which may be related to skeletal abnormalities found in *CHAMP1*^−/−^ mice on mixed background (Supplementary Fig. 2C). Expression changes of most of the DEGs found in *CHAMP1*^−/−^ embryonic brain were not significant in *CHAMP1^+/−^* embryonic brain, but they showed similar expression changes, which was demonstrated by a correlation analysis (Fig. 7C).

To address the relationship of CHAMP1 with ID, we performed GSEA against the 94 genes, in which damaging *de novo* mutations were found in NDD.^6^ The expression of these genes was significantly downregulated not only in *CHAMP1*^−/−^, but also in *CHAMP1^+/−^* embryonic brain (Fig. 7D). We judged significant enrichment of NDD-related genes by testing the gene set together with 1000 random gene sets with comparable sizes (Supplementary Fig. 11B). Collectively, these data suggest that CHAMP1 plays a role in the expression of neurotransmitter transport and NDD-related genes, dysregulation of which may be related to ID.

## Discussion

In this study, we report the first characterization of a *CHAMP1* knockout mouse. We found that *CHAMP1*^−/−^ mice are smaller in size and neonatally lethal. Mitotic cells were increased in *CHAMP1* knockout embryonic cerebral cortex, and *CHAMP1*^−/−^ NSCs showed a delay in neuronal differentiation *in vitro*, which was also suggested *in vivo* by CHAMP1 knockdown. In a behavioural test battery, *CHAMP1^+/−^* mice exhibited mild memory defects, altered social interaction, and depression-like behaviours. Transcriptomic analysis revealed downregulation of neurotransmitter transport and NDD-related genes.

Newborn *CHAMP1*^−/−^ mice were neonatally lethal but often survived the first day after birth. As milk was detected in their stomach (data not shown), severe breathing and suckling problems are unlikely to be the cause of death. Apparent defects in organ structures were not found in *CHAMP1*^−/−^ mice through systemic analysis, leaving the cause of neonatal death of *CHAMP1*^−/−^ mice unspecified. Interestingly, *CHAMP1*^−/−^ mice can survive to adulthood on mixed genetic background, suggesting that the neonatal lethality is somehow rescued on the mixed background.

*CHAMP1* deficiency in NSCs resulted in delayed neuronal differentiation, suggesting that CHAMP1 plays a role in neuronal development. Similar neuronal developmental delay was also reported in NSCs depleted of POGZ,^49^ which may reflect the close relationship between them. Mitotic cells were increased in the cerebral cortex of *CHAMP1* knockout mice. CHAMP1 functions in the regulation of kinetochore–microtubule attachment,^11^ and defective chromosome segregation and multipolar spindle formation were seen in lymphoblast cells isolated from an individual with a *CHAMP1* mutation.^12^ It is known that genes related to spindle formation and chromosome segregation are mutated in severe ID and/or microcephaly, causing cortical development failure.^55^ Therefore, mitotic defects caused by *CHAMP1* deficiency may lead to delay in mitotic progression and neuronal development. However, the delay is supposed to be small and transiently detectable, as impairment of cortical layering was not detected in newborn mice.

Recently, a comprehensive evaluation of the neurobehavioural phenotype of individuals with *CHAMP1* mutations was reported.^10^ In our behavioural test battery, *CHAMP1^+/−^* mice showed a relatively mild phenotype in a subset of tests. However, a slightly cued memory impairment in the cued and contextual fear conditioning test, as well as a slight working memory impairment in the *T*-maze test are consistent with ID, as memory deficits are commonly seen in mouse models of ID and ASD genes, such as *Fmr1*, *Shank* and *Mecp2*.^4^ In addition, altered social interaction, which was found in the 24 h home-cage monitoring test, is also a common feature of NDD.^56^ It is of note that depression-like behaviour was found in *CHAMP1^+/−^* mice in a Porsolt forced swim test. Abnormal findings in a rotarod test may also be related to depression-like behaviour. Interestingly, anxiety and depression were frequently manifested in individuals with *CHAMP1* mutations,^10^ suggesting that depression-like behaviour is a characteristic phenotype of *CHAMP1* deficiency. Pathophysiological significance of increased PPI in *CHAMP1^+/−^* mice is currently unknown. PPI is a common psychophysiological index of sensorimotor gating, and reduction of PPI is typically seen in schizophrenia and also in other disorders.^57^ On the other hand, relationship between increased PPI levels and diseases are relatively unfamiliar. It is noteworthy that mice mutated in *CHD8*, a gene affected in ASD, also exhibit an increased PPI level,^58^ implying a relationship between increased PPI and NDD.

To understand the role of CHAMP1, findings on POGZ, an interaction partner of CHAMP1 that is also related to ID and ASD,^18–21^ are informative. Similarly to CHAMP1, depletion of POGZ was reported to cause a chromosome segregation defect.^59^ POGZ is also involved in DNA double-strand break (DSB) repair,^60^ and we recently reported that CHAMP1 functions in DSB repair together with POGZ.^53^ POGZ is widely expressed in neurons throughout the brain.^25,49,50^  *POGZ* homozygous knockout mice die during embryonic development or shortly after birth.^61,62^ To assess neuronal defects by avoiding embryonic lethality, a brain-specific conditional *POGZ* knockout (*POGZ* cKO) mouse was generated.^50^ In behavioural tests, *POGZ* cKO^−/−^ mice showed motor learning deficits and increased social interaction.^50^ Notably, significant differences were only seen between wild-type and *POGZ* cKO^−/−^ mice but not between wild-type and *POGZ* cKO^+*/−*^ mice, implying that *CHAMP1* deficiency phenotype may become more apparent in a brain-specific conditional *CHAMP1* homozygous knockout mouse. In contrast, a model mouse heterozygous for a *de novo POGZ* mutation found in an ASD patient (*POGZ*^WT/Q1038R^) showed NDD-related behavioural abnormalities, suggesting a dominant-negative effect of the POGZ mutant.^49^ Reduced body weight seen in *CHAMP1* knockout mice was also observed in both *POGZ* cKO and *POGZ*^WT/Q1038R^ mice.^49,50^ Both studies, as well as other studies suggest that POGZ plays a role in transcriptional regulation.^49,50,61,63^ The possibility that CHAMP1 also functions in transcriptional regulation is worth considering, which can explain the downregulation of neurotransmitter transport and NDD-related genes in embryonic *CHAMP1*^−/−^ brains.

It has not been shown whether truncated *CHAMP1* transcripts are expressed in individuals with *CHAMP1* mutations. Even if they are expressed, they will not function properly considering that truncated CHAMP1 proteins cannot localize to chromatin to form a complex with POGZ.^8^ This suggests that individuals with *CHAMP1* mutations show haplo-insufficient phenotype. The observation that severity of ID and other symptoms is not correlated to the size of truncation supports this notion.^8^ However, relatively mild phenotypes reported in individuals with 13q34 microdeletions that include the *CHAMP1* locus^16^ leaves the possibility that truncated CHAMP1 plays a dominant role in the pathogenesis of *CHAMP1* individuals. Further study is required to obtain insights into the pathophysiology of the disorders in humans with *CHAMP1* mutations and to develop therapeutic interventions.

## Supplementary Material

fcac220_Supplementary_Data

## Abbreviations

ADHD =: attention deficit hyperactivity disorder
ASD =: autism spectrum disorder
CAMP =: chromosome alignment-maintaining phosphoprotein
CDK1 =: cyclin-dependent kinase 1
CP =: cortical plate
CS =: conditioned stimulus
DAPI =: 4′,6-diamidino-2-phenylindole
DEG =: differentially expressed gene
DSB =: double-strand break
FC =: fold change
FDR =: false discovery rate
GFAP =: glial fibrillary acidic protein
GFP =: green fluorescent protein
GO =: gene ontology
GSEA =: gene set enrichment analysis
IB =: immunoblot
ID =: intellectual disability
IF =: immunofluorescence
IZ =: intermediate zone
MEF =: mouse embryonic fibroblast
NDD =: neurodevelopmental disorder
NSC =: neural stem cell
POGZ =: pogo transposable element-derived protein with zinc-finger domain
PPI =: prepulse inhibition
SVZ =: sub-ventricular zone
UCS =: unconditioned stimulus
VZ =: ventricular zone
