# Supplementary material for: Deficiency of CHAMP1, a gene related to intellectual disability, causes impaired neuronal development and a mild behavioural phenotype
Source: Brain Commun. 2022 Aug 30;4(5):fcac220. doi: 10.1093/braincomms/fcac220 (PMC9465530; doi:10.1093/braincomms/fcac220)
Supplement: fcac220_Supplementary_Data [file fcac220_supplementary_data.pdf]

Supplementary information

Deficiency of *CHAMP1*, a gene related to intellectual disability, causes impaired neuronal development and a mild behavioral phenotype

Masayoshi Nagai, Kenji Iemura, Takako Kikkawa, Sharmin Naher, Satoko Hattori, Hideo Hagihara, Koh-ichi Nagata, Hayato Anzawa, Risa Kugisaki, Hideki Wanibuchi, Takaya Abe, Kenichi Inoue, Kengo Kinoshita, Tsuyoshi Miyakawa, Noriko Osumi, and Kozo Tanaka

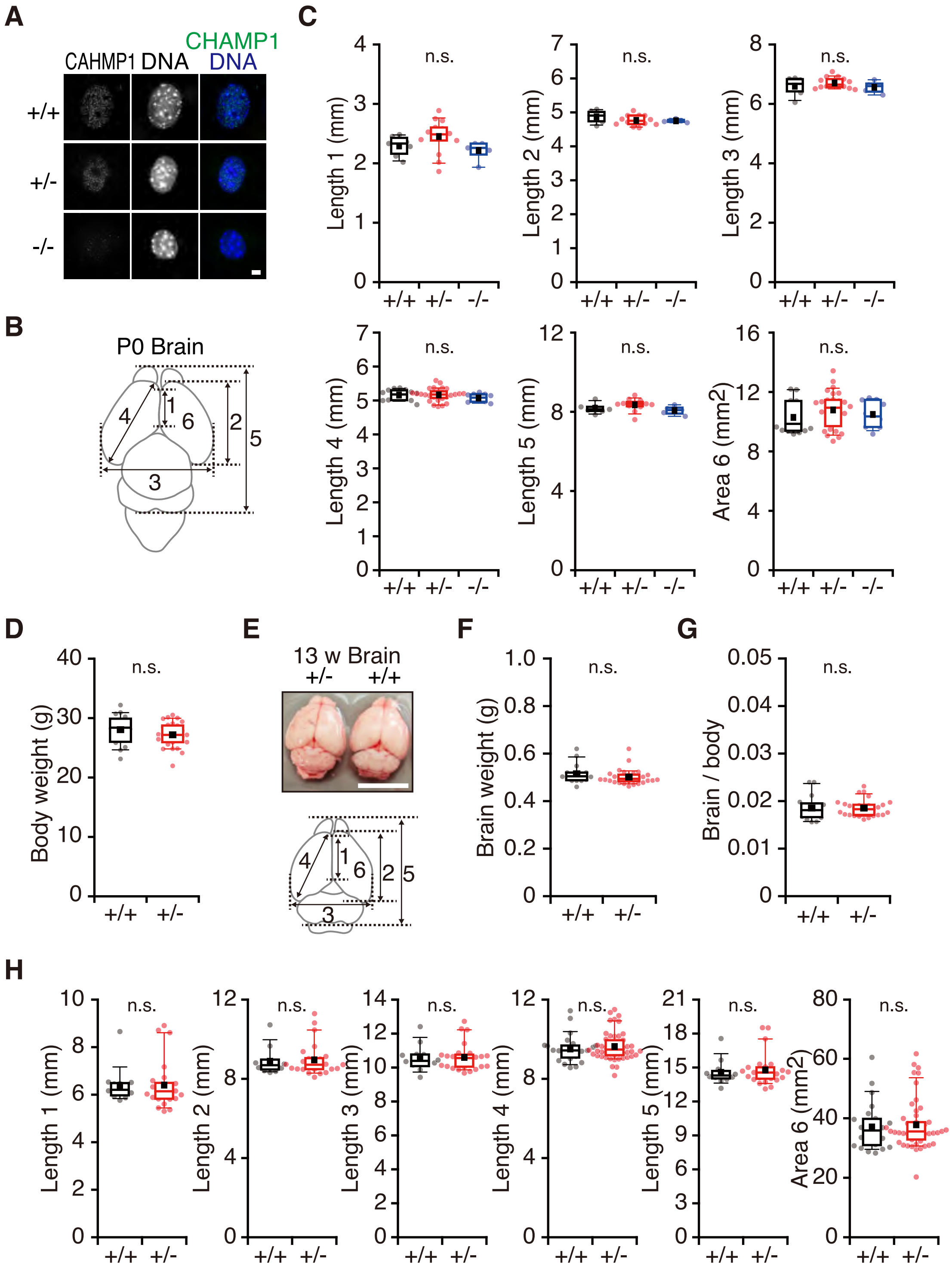

A
CAHMP1
DNA
CHAMP1
DNA
+/+
+/-
-/-
B
P0 Brain
C
n.s.
Length 1 (mm)
Length 2 (mm)
Length 3 (mm)
Length 4 (mm)
Length 5 (mm)
Area 6 (mm2)
D
Body weight (g)
E
13 w Brain
F
Brain weight (g)
G
Brain / body
H

Supplementary Figure 1. ***CHAMP1$^{-/-}$*** **mice do not show gross defects in brain size.** (A) Immunofluorescence analysis of mouse embryonic fibroblasts (MEFs) isolated from *CHAMP1$^{+/+}$*, *CHAMP1$^{+/-}$*, and *CHAMP1$^{-/-}$* mice using an antibody against CHAMP1 (green). DNA was stained with DAPI (blue). Scale bar: 5 μm. (B) Schematic diagrams of newborn mouse brains indicating cortical parameters measured in C. (C) Anteroposterior length (shown as 1 in (B)), cortex length (shown as 2 in (B)), cortex width (shown as 3 in (B)), cortex length 2 (shown as 4 in (B)), brain length (shown as 5 in (B)), and cortex area (shown as 6 in (B)) of *CHAMP1$^{+/+}$* (black), *CHAMP1$^{+/-}$* (red), and *CHAMP1$^{-/-}$* (blue) mouse brains. (1-3, 5) *CHAMP1$^{+/+}$*; n = 7, *CHAMP1$^{+/-}$*; n = 14, *CHAMP1$^{-/}$*; n = 5. (4, 6) *CHAMP1$^{+/+}$*; n = 14, *CHAMP1$^{+/-}$*; n = 28, *CHAMP1$^{-/-}$*; n = 10. (D) Absolute body weight of 13 week-old mice (*CHAMP1$^{+/+}$* (black); n = 16, *CHAMP1$^{+/-}$* (red); n = 29). (E) Representative pictures of 13 week-old *CHAMP1$^{+/+}$* and *CHAMP1$^{+/-}$* mouse brains and schematic diagrams of adult mouse brains indicating cortical parameters measured in H. Scale bar: 1 cm. (F, G) Absolute brain weight (F) and ratios of brain weight to body weight (G) of 13 week-old mice (*CHAMP1$^{+/+}$* (black); n = 16, *CHAMP1$^{+/-}$* (red); n = 29). (H) Anteroposterior length (shown as 1 in (E)), cortex length (shown as 2 in (E)), cortex width (shown as 3 in (E)), cortex length 2 (shown as 4 (E)), brain length (shown as 5 in (B), and cortex area (shown as 6 in (B)) of 13 week-old *CHAMP1$^{+/+}$* (black) and *CHAMP1$^{+/-}$* (red) mouse brains. (1-3, 5) *CHAMP1$^{+/+}$*; n = 12, *CHAMP1$^{+/-}$*; n = 25. (4, 6) *CHAMP1$^{+/+}$*; n = 24, *CHAMP1$^{+/-}$*; n = 50. In box plots, the bottom and top of the box show the lower and upper quartile values, respectively. The mean is indicated with a filled square and the median is indicated with a bar in the box. The bottom and top of the whiskers denote the 10th and 90th percentiles, respectively. To judge whether the differences observed were statistically significant, *p* values for genotype effects were determined either by Kruskal–Wallis test. *n.s.*, not statistically significant.

A

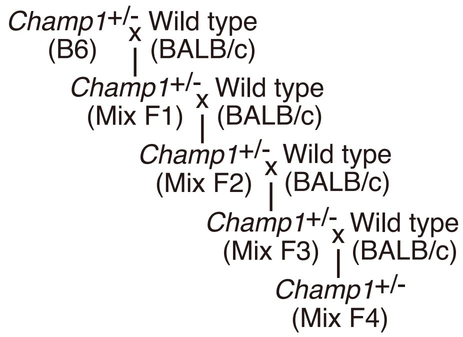

B

+/- x +/-	+/+	+/-	-/-
Mix F1 x F1	42 (1.00)	85 (2.02)	16 (0.38)
Mix F2 x F2	33 (1.00)	66 (2.00)	6 (0.18)
Mix F3 x F3	24 (1.00)	40 (1.67)	0
Mix F4 x F4	23 (1.00)	44 (1.91)	0

C

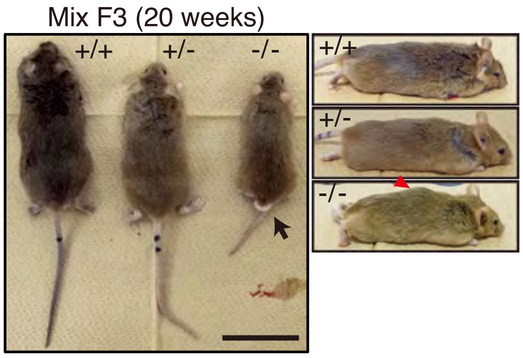

D

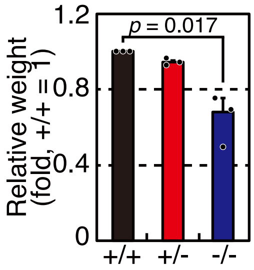

E

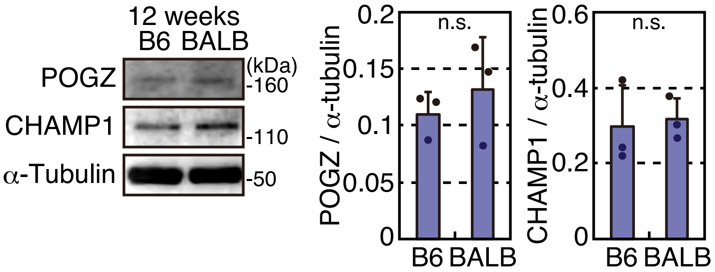

Supplementary Figure 2. *CHAMP1$^{-/-}$* mice can survive to adulthood on mixed genetic background. (A) Procedure to produce *CHAMP1* knockout mice on mixed genetic background. (B) Number and ratio of *CHAMP1$^{+/+}$*, *CHAMP1$^{+/-}$*, *CHAMP1$^{-/-}$* (blue) mice from crosses of *CHAMP1$^{+/-}$* mice on respective mixed backgrounds as shown in (A). (C) (left) Representative picture of 20 week-old *CHAMP1$^{+/+}$*, *CHAMP1$^{+/-}$*, and *CHAMP1$^{-/-}$* mice on mixed background (F3). Scale bar: 5 cm. (right) Side view of the mice shown in the left panel. Kinked tail and kyphosis in a *CHAMP1$^{-/-}$* mouse are shown by black arrows and a red arrowhead, respectively. (D) Comparison of the body weight in newborn mice (*CHAMP1$^{+/+}$* (black); n = 3, *CHAMP1$^{+/-}$* (red); n = 3, *CHAMP1$^{+/-}$* (blue); n = 3) on mixed background (F3). Error bars represent S.D. *p* values were determined by Student's *t*-test. (E) Expression of CHAMP1 and POGZ in C57BL/6 and Balb/c mice. Whole-cell lysates prepared from 12 week-old mouse brains were subjected to immunoblot analysis using antibodies as indicated. Quantification results of CHAMP1 and POGZ are shown on the right (n = 3). Error bars represent S.D. *p* values were determined by Student's t-test. n.s., not statistically significant.

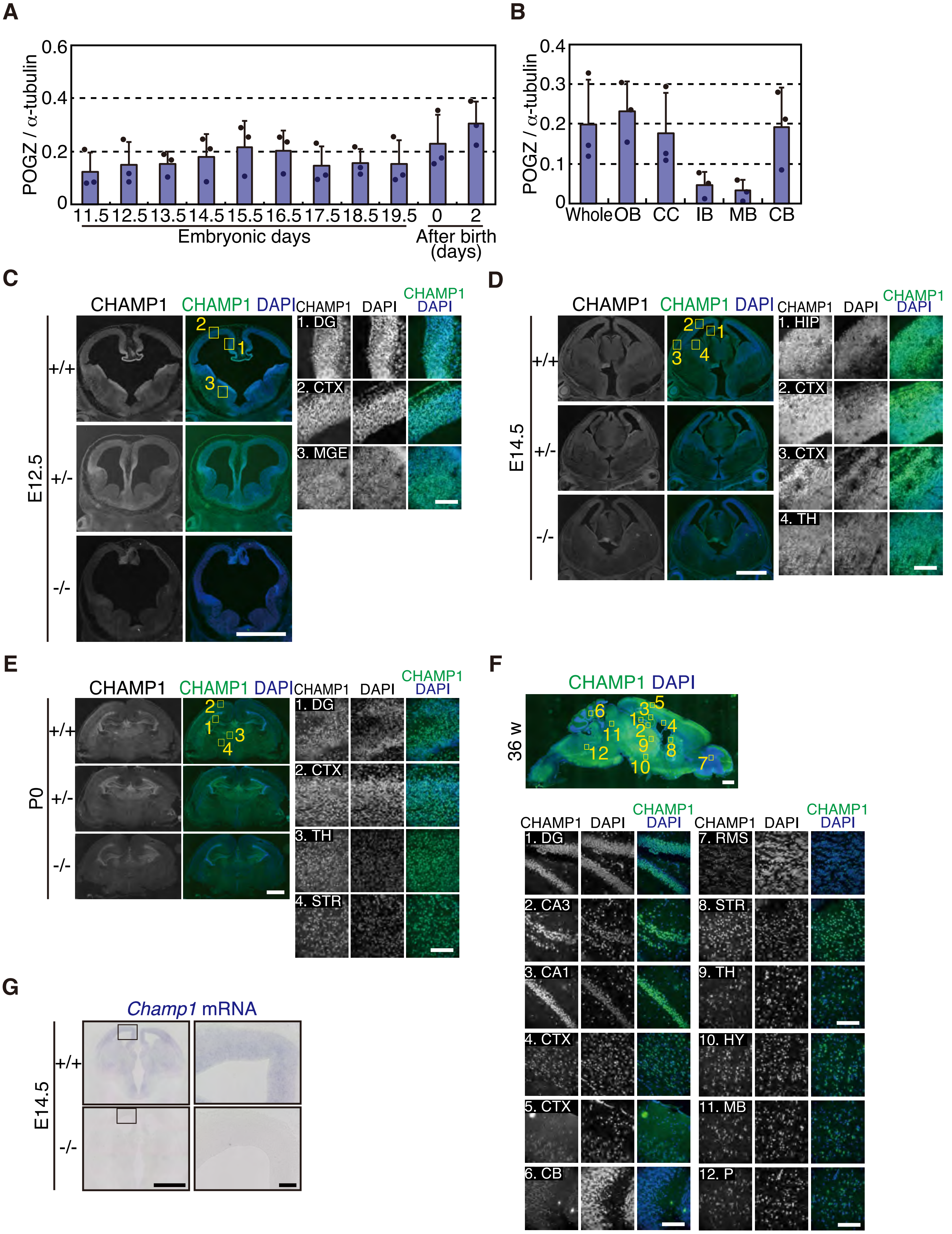
A
POGZ / α-tubulin
0.6
0.4
0.2
0
11.5 12.5 13.5 14.5 15.5 16.5 17.5 18.5 19.5
Embryonic days
0 2
After birth (days)
B
POGZ / α-tubulin
0.4
0.3
0.2
0.1
0
Whole OB CC IB MB CB
C
CHAMP1
CHAMP1 DAPI
E12.5
+/+
+/-
-/-
1. DG
2. CTX
3. MGE
D
E14.5
1. HIP
2. CTX
3. CTX
4. TH
E
P0
1. DG
2. CTX
3. TH
4. STR
F
CHAMP1 DAPI
36 w
1. DG
2. CA3
3. CA1
4. CTX
5. CTX
6. CB
7. RMS
8. STR
9. TH
10. HY
11. MB
12. P
G
Champ1 mRNA
E14.5
+/+
-/-

Supplementary Figure 3. POGZ and CHAMP1 expression in mouse brains. (A) Quantification results of POGZ in brain during development shown in Fig. 2A (n = 3). (B) Quantification results of POGZ in whole brain, olfactory bulb (OB), cerebral cortex (CC), interbrain (IB), midbrain (MB), and cerebellum (CB) of newborn mice shown in Fig. 2B. (C–E) Immunofluorescence staining of coronal cortical sections from E12.5 (C), E14.5 (D), and newborn (E) *CHAMP1*$^{+/+}$ (upper), *CHAMP1*$^{+/-}$ (middle), and *CHAMP1*$^{-/-}$ (lower) mouse brains using an antibody against CHAMP1 (green). Nuclei were stained with DAPI (blue). (F) Immunofluorescence staining of a sagittal section from a 36 week-old mouse using an antibody against CHAMP1 (green). Nuclei were stained with DAPI (blue). Scale bars: 1 mm. Scale bars in close-up images of boxed area: 100 μm. DG, dentate gyrus; CTX, cerebral cortex; Tel: telencephalon, HIP, hippocampus; TH, thalamus; CA, caudate nucleus; CB, cerebellum; RMS, rostral migratory stream; STR, striatum; HY, hypothalamus; MB, midbrain; P, pons. (G) *In situ* hybridization of *CHAMP1* in coronal cortical sections from E14.5 *CHAMP1*$^{+/+}$ (upper) and *CHAMP1*$^{-/-}$ (lower) mouse brains. Scale bar: 1 mm. Scale bar in close-up images of boxed area: 100 μm.

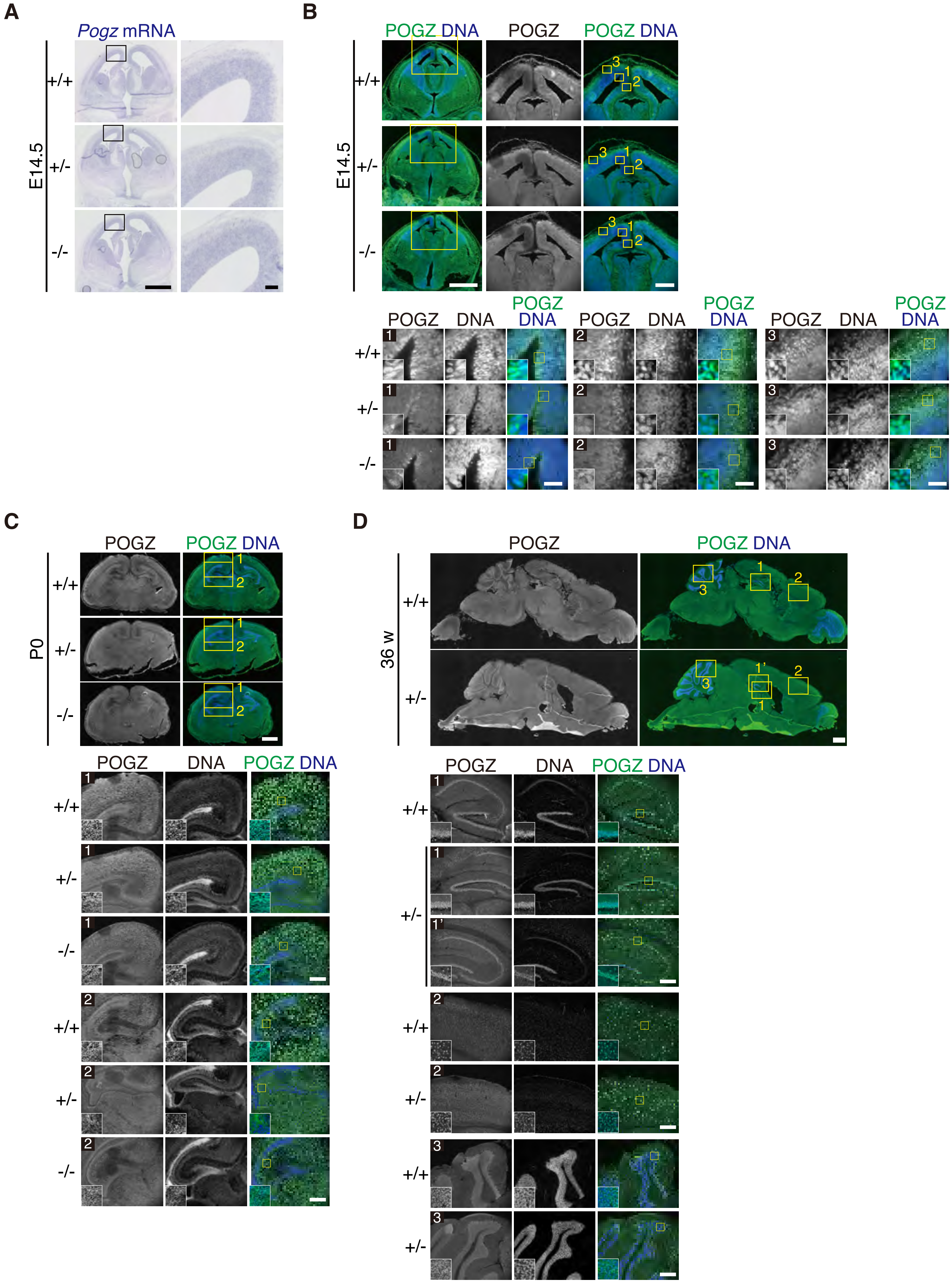

A
Pogz mRNA
E14.5
+/+
+/-
-/-
B
POGZ DNA
POGZ
POGZ DNA
E14.5
+/+
+/-
-/-
1
2
3
POGZ
DNA
POGZ DNA
C
POGZ
POGZ DNA
P0
+/+
+/-
-/-
1
2
POGZ
DNA
POGZ DNA
D
POGZ
POGZ DNA
36 w
+/+
+/-
1
1'
2
3
POGZ
DNA
POGZ DNA

Supplementary Figure 4. POGZ expression in *CHAMP1* knockout mouse brains. (A) *In situ* hybridization of *POGZ* in coronal cortical sections from E14.5 *CHAMP1*$^{+/+}$ (upper), *CHAMP1*$^{+/-}$ (middle), and *CHAMP1*$^{-/-}$ (lower) mouse brains. Scale bar: 1 mm. Scale bar in close-up images of boxed area: 100 μm. (B–D) Immunofluorescence staining of coronal cortical sections from E14.5 (B), newborn (C) *CHAMP1*$^{+/+}$ (upper), *CHAMP1*$^{+/-}$ (middle), and *CHAMP1*$^{-/-}$ (lower) mouse brains, and sagittal sections from (D) *CHAMP1*$^{+/+}$ (upper; 36 week-old) and *CHAMP1*$^{+/-}$ (lower; 37 week-old) mouse brains using an antibody against POGZ (green). Nuclei were stained with DAPI (blue). Scale bars: 1 mm. Scale bars in close-up images of boxed area: 50 μm (B) or 100 μm (C, D).

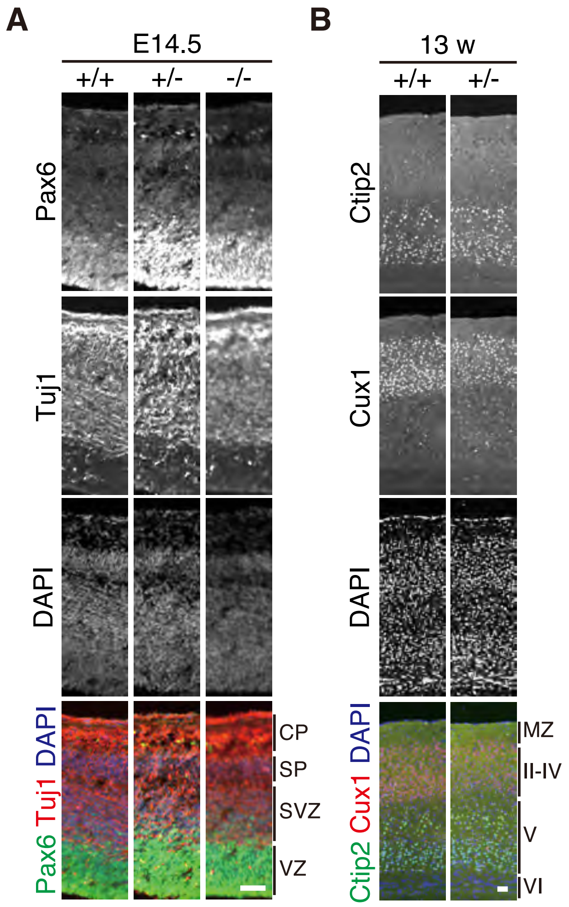
A
E14.5
+/+
+/-
-/-
Pax6
Tuj1
DAPI
Pax6 Tuj1 DAPI
CP
SP
SVZ
VZ
B
13 w
+/+
+/-
Ctip2
Cux1
DAPI
Ctip2 Cux1 DAPI
MZ
II-IV
V
VI

Supplementary Figure 5. ***CHAMP1*$^{-/-}$ mice do not show gross defects in brain structure.** (A) Immunofluorescence staining of a large dorsal cortical region of E14.5 *CHAMP1*$^{+/+}$, *CHAMP1*$^{+/-}$, and *CHAMP1*$^{-/-}$ mice using antibodies against Pax6 (green) and Tuj1 (red). Nuclei were stained with DAPI (blue). Scale bar: 50 μm. (B) Immunofluorescence staining of a large dorsal cortical region of 35 week-old *CHAMP1*$^{+/+}$ and *CHAMP1*$^{+/-}$ mice using antibodies against Ctip2 (green) and Cux1 (red). Nuclei were stained with DAPI (blue). Scale bars: 50 μm. CP, cortical plate; SP, sub-plate; SVZ, sub-ventricular zone; VZ, ventricular zone; II～VI, cortical layers.

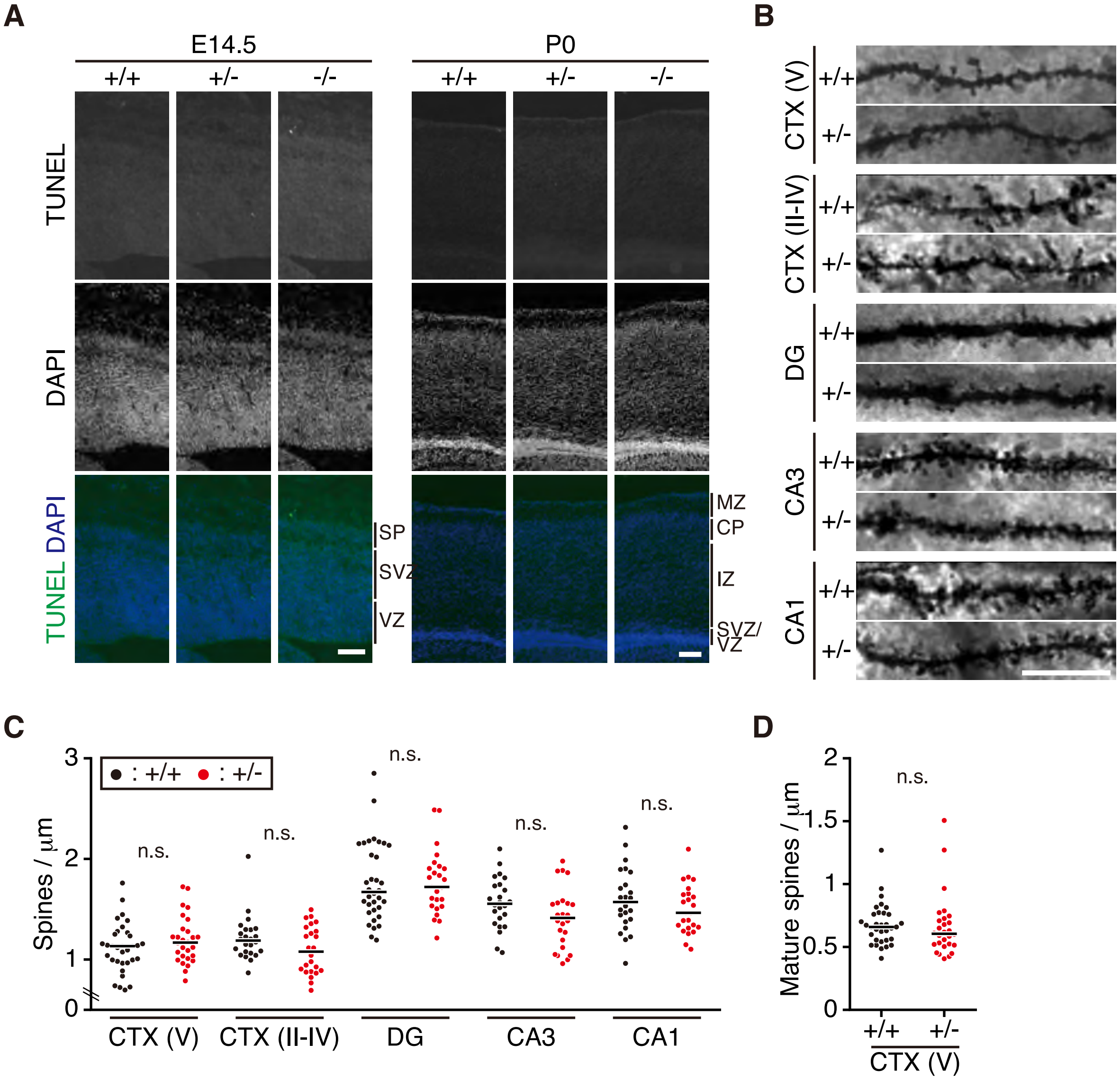
A
E14.5
P0
+/+
+/-
-/-
TUNEL
DAPI
TUNEL DAPI
SP
SVZ
VZ
MZ
CP
IZ
SVZ/
VZ
B
CTX (V)
CTX (II-IV)
DG
CA3
CA1
C
: +/+
: +/-
Spines / μm
n.s.
CTX (V)
CTX (II-IV)
DG
CA3
CA1
D
Mature spines / μm
n.s.
CTX (V)

Supplementary Figure 6. TUNEL staining and Golgi–Cox staining of *CHAMP1* knockout mouse brain. (A) TUNEL staining (green) of a large dorsal cortical region of E14.5 or newborn *CHAMP1*$^{+/+}$, *CHAMP1*$^{+/-}$, and *CHAMP1*$^{-/-}$ mice. Nuclei were stained with DAPI (blue). Scale bars: 100 µm. SP, sub-plate; SVZ, sub-ventricular zone; VZ, ventricular zone; MZ, marginal zone; CP, cortical plate; IZ, intermediate zone. (B) Dendrites in cerebral cortex (CTX), dentate gyrus (DG), and caudate nucleus (CA) of 13 week-old *CHAMP1*$^{+/+}$ and *CHAMP1*$^{+/-}$ mice visualized by Golgi–Cox staining. Scale bar: 10 µm. (C) Dendritic spine density in 13 week-old mouse brains (*CHAMP1*$^{+/+}$: CTX (V); n = 33, CTX (II-IV); n = 24, DG; n = 34, CA3; n = 24, CA1; n = 24, *CHAMP1*$^{+/-}$: CTX (V); n = 28, CTX (II-IV); n = 24, DG; n = 25, CA3; n = 24, CA1; n = 24). The median is indicated with a bar. *p* values were determined by the Mann–Whitney *U*-test. n.s., not statistically significant. (D) Mature spine density in layer 5 of cerebral cortex of 13 week-old mice (*CHAMP1*$^{+/+}$: CTX (V); n = 33, *CHAMP1*$^{+/-}$: CTX (V); n = 28). The median is indicated with a bar. *p* value was determined by the Mann–Whitney *U*-test. n.s., not statistically significant.

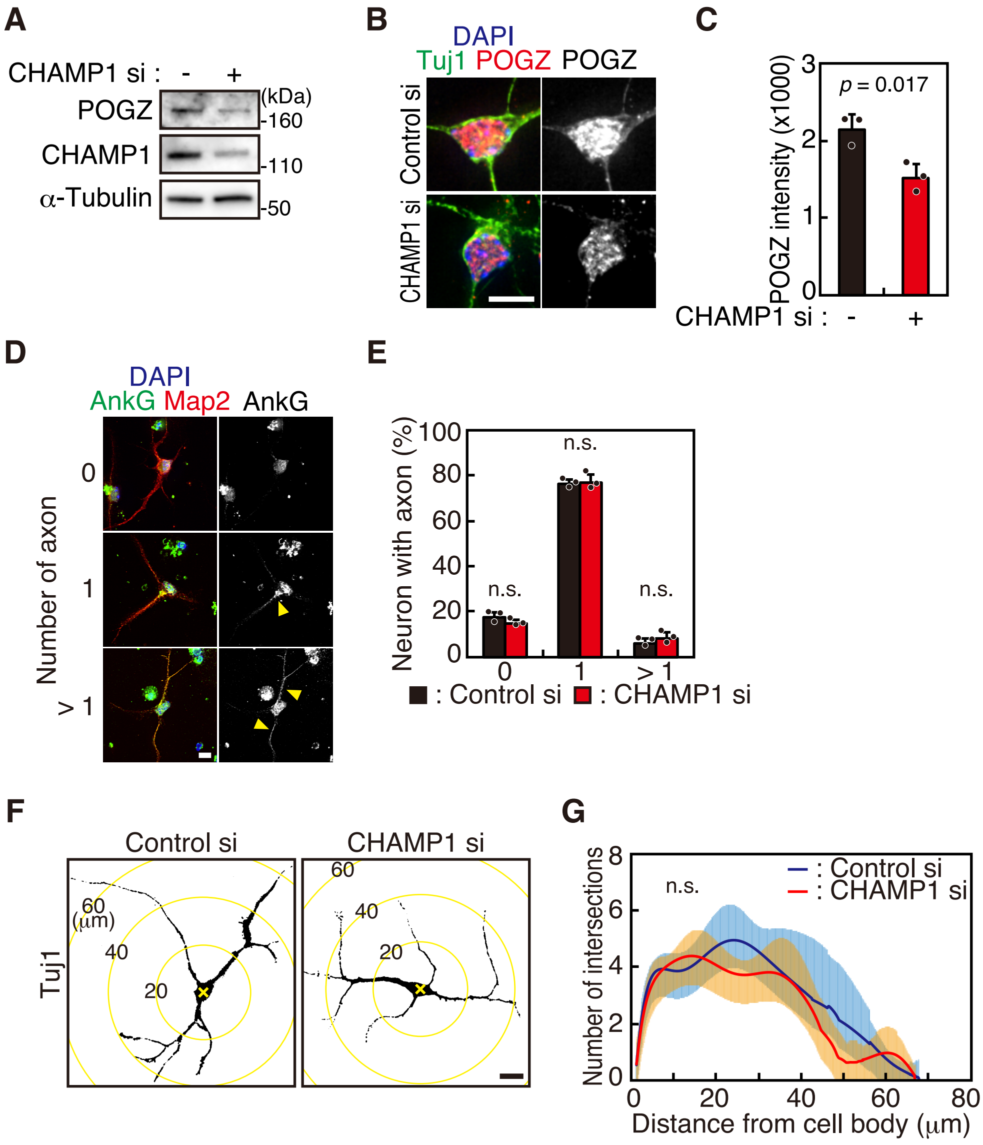

A
CHAMP1 si : - +
POGZ
CHAMP1
α-Tubulin
(kDa)
-160
-110
-50
B
DAPI
Tuj1 POGZ POGZ
Control si
CHAMP1 si
C
p = 0.017
POGZ intensity (x1000)
CHAMP1 si : - +
D
DAPI
AnkG Map2 AnkG
Number of axon
0
1
> 1
E
Neuron with axon (%)
n.s.
: Control si : CHAMP1 si
F
Control si
CHAMP1 si
Tuj1
60 (μm)
40
20
G
n.s.
: Control si
: CHAMP1 si
Number of intersections
Distance from cell body (μm)

Supplementary Figure 7. Axon outgrowth and dendritic branching in CHAMP1 knockdown neurons. (A) *CHAMP1* RNAi in neurons. Neurospheres transfected with a *CHAMP1* siRNA were differentiated for five days and subjected to immunoblot analysis using antibodies as indicated. (B) POGZ localization in control or CHAMP1 knockdown neurons. Neurospheres transfected with a *CHAMP1* siRNA were differentiated for five days and immunostained with antibodies against Tuj1 (green) and POGZ (red). Nuclei were stained with DAPI (blue). Scale bar: 10 μm. (C) Signal intensity of POGZ immunofluorescence in control or CHAMP1 knockdown neurons (n = 3 mice, data represent average intensity from 10 cells per mouse). Error bars represent S.D. *p* values were determined by Student's t-test. (D) Immunofluorescence staining of neurons differentiated from neurospheres with different number of axons using antibodies against Map2 (red) and Ankyrin-G (green). Nuclei were stained with DAPI (blue). Scale bar: 10 μm. (E) Number of axons in CHAMP1 knockdown neurons treated as in (D). The data represent a minimum of 81 cells for each condition. Error bars represents S.D. *p* values were determined by Tukey–Kramer test. n.s., not statistically significant. (F) Representative images of dendritic branching in control or CHAMP1 knockdown neurons differentiated from neurospheres. Distance (in μm) from the center of the cell body, shown as "X", is indicated for Sholl analysis. (G) Quantification of the number of intersections of control or CHAMP1 knockdown neurons differentiated from neurospheres at various distances from the cell body by Sholl analysis (n = 6 differentiated neurons). S.D. is indicated as shaded area. *p* values were determined by Tukey–Kramer test. n.s., not statistically significant.

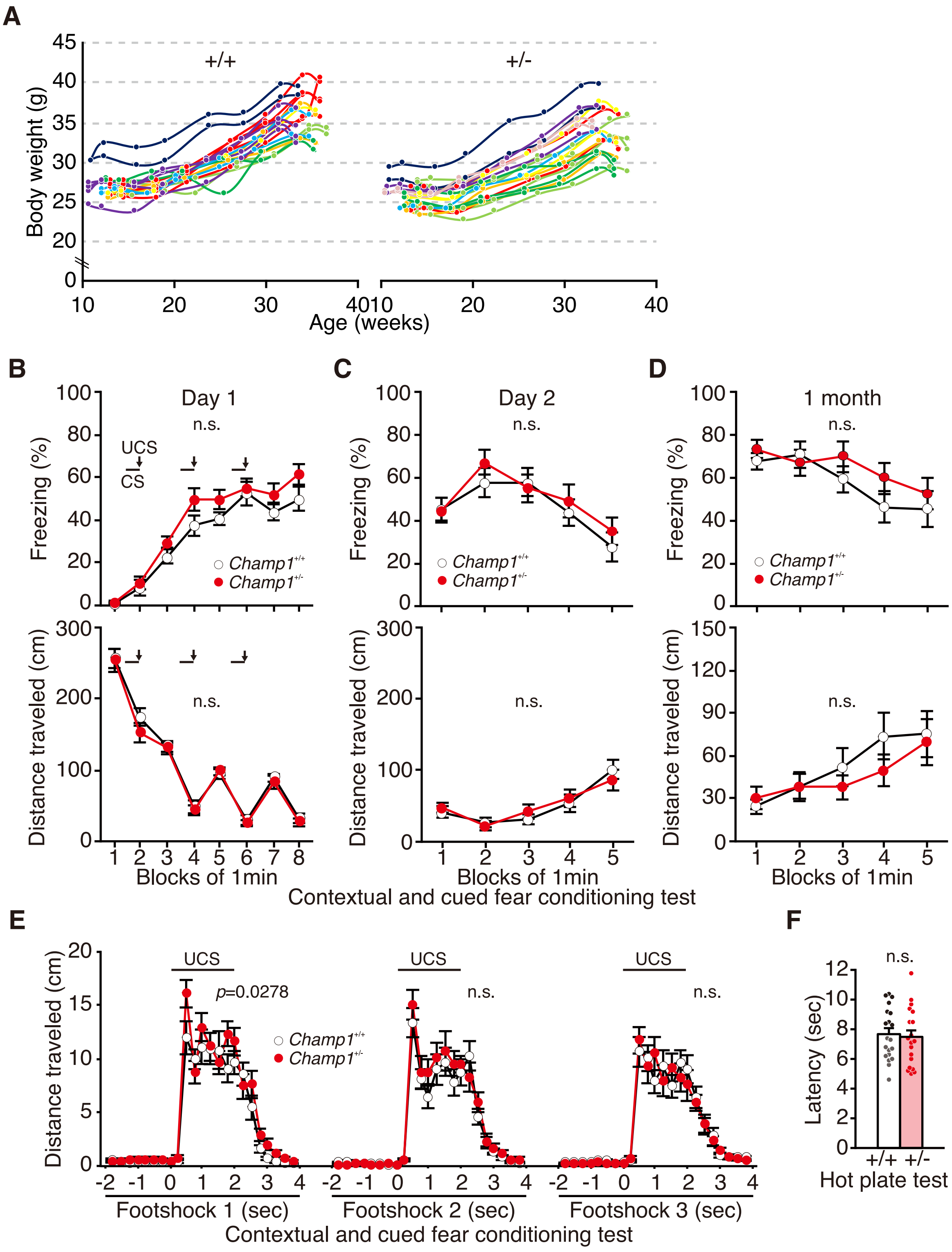

A
+/+
+/-
Body weight (g)
Age (weeks)
B
Day 1
n.s.
UCS
CS
Freezing (%)
Champ1+/+
Champ1+/-
Distance traveled (cm)
Blocks of 1min
C
Day 2
D
1 month
Contextual and cued fear conditioning test
E
UCS
p=0.0278
Footshock 1 (sec)
Footshock 2 (sec)
Footshock 3 (sec)
F
Latency (sec)
Hot plate test

Supplementary Figure 8. Body weight, contextual and cued fear conditioning test, and hot plate test in *CHAMP1*$^{+/-}$ mice. (A) Body weight of *CHAMP1*$^{+/+}$ (+/+; n = 20) and *CHAMP1*$^{+/-}$ (+/-; n = 20) mice during the observation period of the behavioral test battery, plotted against age in weeks. Littermates are shown in the same colors. (B–E) Contextual and cued fear conditioning test: (B) percentage of freezing and total distance traveled in conditioning on day 1, (C) percentage of freezing and total distance traveled in context testing on day 2, (D) percentage of freezing and total distance traveled in context testing after 1 month, and (E) distance traveled during and after each foot shock in conditioning on day 1 of *CHAMP1*$^{+/+}$ (white circle) and *CHAMP1*$^{+/-}$ (red circle) mice. CS (conditioned stimulus): 55 dB white noise (30 sec). UCS (unconditioned stimulus): 0.3 mA foot shock (2 sec). (F) Latency to the first hind paw response of *CHAMP1*$^{+/+}$ (white bar) and *CHAMP1*$^{+/-}$ (red bar) mice in the hot-plate test. For (B-F), twenty male mice of both genotypes were used. Error bars represent S.E.M. To judge whether the differences observed were statistically significant, *p* values for genotype effects were determined either by two-way repeated measurement ANOVA (B–E) or Student's *t*-test (F).

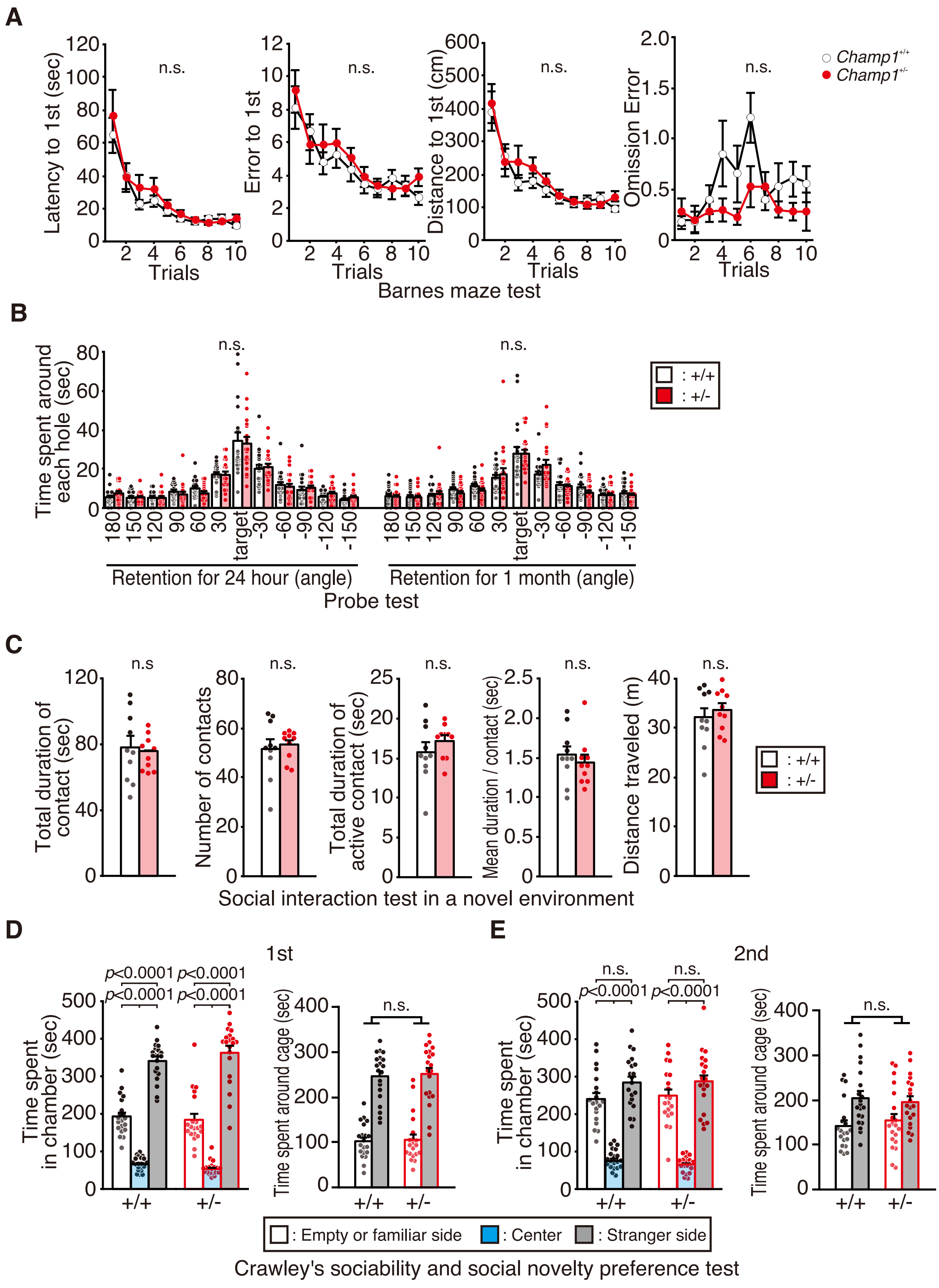
A
Latency to 1st (sec)
Error to 1st
Distance to 1st (cm)
Omission Error
Trials
n.s.
Champ1+/+
Champ1+/-
Barnes maze test
B
Time spent around each hole (sec)
n.s.
: +/+
: +/-
Retention for 24 hour (angle)
Retention for 1 month (angle)
Probe test
C
Total duration of contact (sec)
Number of contacts
Total duration of active contact (sec)
Mean duration / contact (sec)
Distance traveled (m)
Social interaction test in a novel environment
D
1st
Time spent in chamber (sec)
Time spent around cage (sec)
p<0.0001
E
2nd
: Empty or familiar side
: Center
: Stranger side
Crawley's sociability and social novelty preference test

Supplementary Figure 9. Barnes maze test, social interaction test, and Crawley's sociability and social novelty preference test in *CHAMP1*$^{+/-}$ mice. (A, B) Barnes maze test: (A) latency to reach the target, number of errors until reaching the target, total distance traveled to reach the target, and number of errors to enter the escape box after reaching the target for *CHAMP1*$^{+/+}$ (white circle) and *CHAMP1*$^{+/-}$ (red circle) mice and (B) time spent around each hole in the first and the second probe test by *CHAMP1*$^{+/+}$ (white bars) and *CHAMP1*$^{+/-}$ (red bars) mice. (C) Social interaction test: total duration of contact, number of contacts, total duration of active contact, mean duration of each contact, and total distance traveled of *CHAMP1*$^{+/+}$ (white bar) and *CHAMP1*$^{+/-}$ (red bar) mice. (D, E) Crawley's sociability and social novelty preference test: (D) left graph: time spent in the chamber with an empty cage (white bars), the center chamber (light blue bars), or the chamber with a cage containing a stranger mouse (stranger 1, gray bars) during the first task, right graph: time spent around empty (white bars) or stranger (black bars) cage during the first task, by *CHAMP1*$^{+/+}$ (black dots) and *CHAMP1*$^{+/-}$ (red dots) mice. (E) left graph: time spent in the chamber with the cage containing stranger 1 (white bars), the center chamber (light blue bars), or the chamber with a cage containing a novel unfamiliar mouse (stranger 2, gray bars) during the second task, right graph: time spent around familiar (white bars) or stranger (black bars) cage during the second task, by *CHAMP1*$^{+/+}$ (black dots) and *CHAMP1*$^{+/-}$ (red dots) mice. Error bars represent S.E.M. *p* values for genotype effects were determined either by two-way repeated measurement ANOVA (A, B), Student's *t*-test (C), and paired *t*-test (D, E).

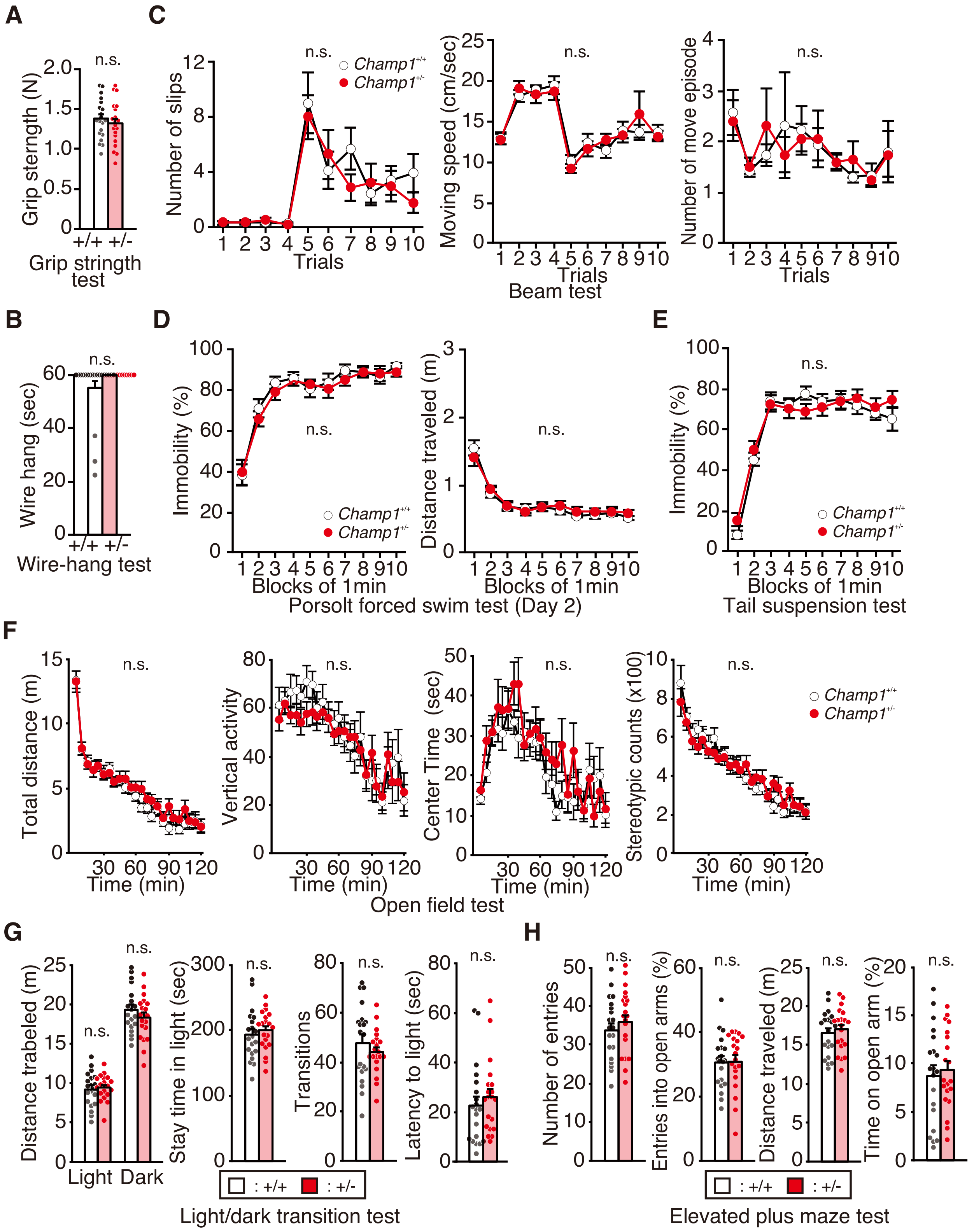
A
Grip sterngth (N)
n.s.
+/+ +/-
Grip stringth test
B
Wire hang (sec)
n.s.
+/+ +/-
Wire-hang test
C
Number of slips
n.s.
Champ1+/+
Champ1+/-
Trials
Moving speed (cm/sec)
Trials
Number of move episode
Trials
Beam test
D
Immobility (%)
Blocks of 1min
Distance traveled (m)
Blocks of 1min
Porsolt forced swim test (Day 2)
E
Immobility (%)
Blocks of 1min
Tail suspension test
F
Total distance (m)
Time (min)
Vertical activity
Center Time (sec)
Stereotypic counts (x100)
Open field test
G
Distance trabeled (m)
Light Dark
Stay time in light (sec)
Transitions
Latency to light (sec)
: +/+ : +/-
Light/dark transition test
H
Number of entries
Entries into open arms (%)
Distance traveled (m)
Time on open arm (%)
Elevated plus maze test

Supplementary Figure 10. Grip strength test, wire-hang test, beam test, Porsolt forced swim test, tail suspension test, open field test, light/dark transition test, and elevated plus maze test in *CHAMP1*$^{+/-}$ mice. (A) Grip strength of *CHAMP1*$^{+/+}$ (white bar) and *CHAMP1*$^{+/-}$ (red bar) mice. (B) Latency to fall of *CHAMP1*$^{+/+}$ (white bar) and *CHAMP1*$^{+/-}$ (red bar) mice in the wire-hang test. (C) Beam test: number of slips, moving speed, and number of move episode for *CHAMP1*$^{+/+}$ (white circle) and *CHAMP1*$^{+/-}$ (red circle) mice. (D) Porsolt forced swim test: percentage of immobility and total distance traveled of *CHAMP1*$^{+/+}$ (white circle) and *CHAMP1*$^{+/-}$ (red circle) mice on day 2. (E) Percentage of immobility of *CHAMP1*$^{+/+}$ (white circle) and *CHAMP1*$^{+/-}$ (red circle) mice in the tail suspension test. (F) Open field test: distance traveled, vertical activity, center time, and stereotypic counts for each 5-minute block of testing of *CHAMP1*$^{+/+}$ (white circle) and *CHAMP1*$^{+/-}$ (red circle) mice. (G) Light/dark transition test: distance traveled in the light and dark chambers, time spent in the light chamber, number of transitions, and latency to enter the light chamber of *CHAMP1*$^{+/+}$ (white bars) and *CHAMP1*$^{+/-}$ (red bars) mice. (H) Elevated plus maze test: number of arm entries, entries into open arms, distance traveled, and time spent in open arms of *CHAMP1*$^{+/+}$ (white bars) and *CHAMP1*$^{+/-}$ (red bars) mice. Twenty male mice of both genotypes were used. Error bars represent S.E.M. p values were determined either by Student's t-test (A, B, G, H) or two-way repeated measurement ANOVA (C–F).

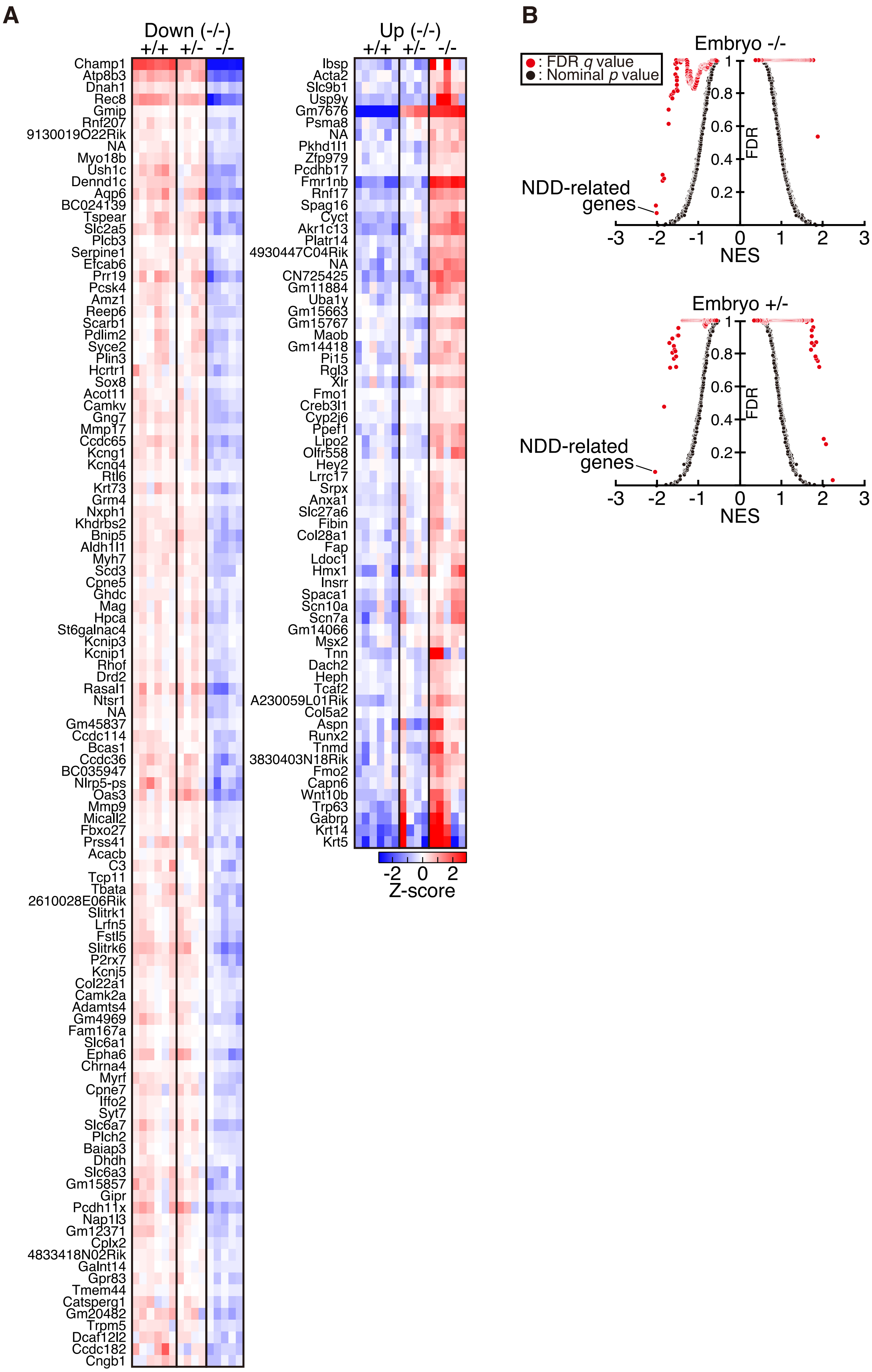
A
Down (-/-)
+/+ +/- -/-
Champ1
Atp8b3
Dnah1
Rec8
Gmip
Rnf207
9130019O22Rik
NA
Myo18b
Ush1c
Dennd1c
Aqp6
BC024139
Tspear
Slc2a5
Plcb3
Serpine1
Efcab6
Prr19
Pcsk4
Amz1
Reep6
Scarb1
Pdlim2
Syce2
Plin3
Hcrtr1
Sox8
Acot11
Camkv
Gng7
Mmp17
Ccdc65
Kcng1
Kcnq4
Rtl6
Krt73
Grm4
Nxph1
Khdrbs2
Bnip5
Aldh1l1
Myh7
Scd3
Cpne5
Ghdc
Mag
Hpca
St6galnac4
Kcnip3
Kcnip1
Rhof
Drd2
Rasal1
Ntsr1
NA
Gm45837
Ccdc114
Bcas1
Ccdc36
BC035947
Nlrp5-ps
Oas3
Mmp9
Micall2
Fbxo27
Prss41
Acacb
C3
Tcp11
Tbata
2610028E06Rik
Slitrk1
Lrfn5
Fstl5
Slitrk6
P2rx7
Kcnj5
Col22a1
Camk2a
Adamts4
Gm4969
Fam167a
Slc6a1
Epha6
Chrna4
Myrf
Cpne7
Iffo2
Syt7
Slc6a7
Plch2
Baiap3
Dhdh
Slc6a3
Gm15857
Gipr
Pcdh11x
Nap1l3
Gm12371
Cplx2
4833418N02Rik
Galnt14
Gpr83
Tmem44
Catsperg1
Gm20482
Trpm5
Dcaf12l2
Ccdc182
Cngb1
Up (-/-)
+/+ +/- -/-
Ibsp
Acta2
Slc9b1
Usp9y
Gm7676
Psma8
NA
Pkhd1l1
Zfp979
Pcdhb17
Fmr1nb
Rnf17
Spag16
Cyct
Akr1c13
Platr14
4930447C04Rik
NA
CN725425
Gm11884
Uba1y
Gm15663
Gm15767
Maob
Gm14418
Pi15
Rgl3
Xlr
Fmo1
Creb3l1
Cyp2j6
Ppef1
Lipo2
Olfr558
Hey2
Lrrc17
Srpx
Anxa1
Slc27a6
Fibin
Col28a1
Fap
Ldoc1
Hmx1
Insrr
Spaca1
Scn10a
Scn7a
Gm14066
Msx2
Tnn
Dach2
Heph
Tcaf2
A230059L01Rik
Col5a2
Aspn
Runx2
Tnmd
3830403N18Rik
Fmo2
Capn6
Wnt10b
Trp63
Gabrp
Krt14
Krt5
-2 0 2
Z-score
B
Embryo -/-
: FDR q value
: Nominal p value
1
0.8
0.6
0.4
0.2
FDR
NDD-related genes
-3 -2 -1 0 1 2 3
NES
Embryo +/-
1
0.8
0.6
0.4
0.2
FDR
NDD-related genes
-3 -2 -1 0 1 2 3
NES

Supplementary Figure 11. Gene expression changes in *CHAMP1* knockout mice. (A) Heatmap visualization of DEGs between wild type, *CHAMP1*$^{+/-}$ and *CHAMP1*$^{-/-}$ embryonic brains with FDR <0.1 and fold change >1.2. (B) Normalized enrichment score (NES) versus FDR *q* value (red dots) and nominal *p* value (black dots) in GSEA between wild type and *CHAMP1*$^{-/-}$ (upper) or *CHAMP1*$^{+/-}$ (lower) embryonic brains for the NDD-related gene set and 1,000 randomly picked gene sets.

Figure 1E

Figure 5A (left)

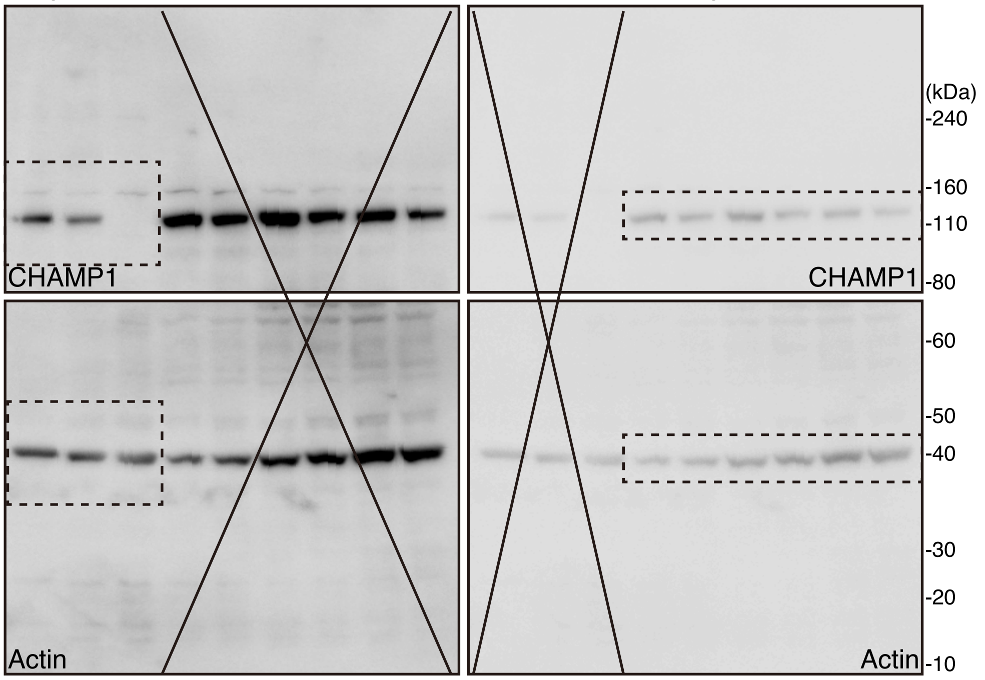

(Long exposure)

(Short exposure)

Figure 2A

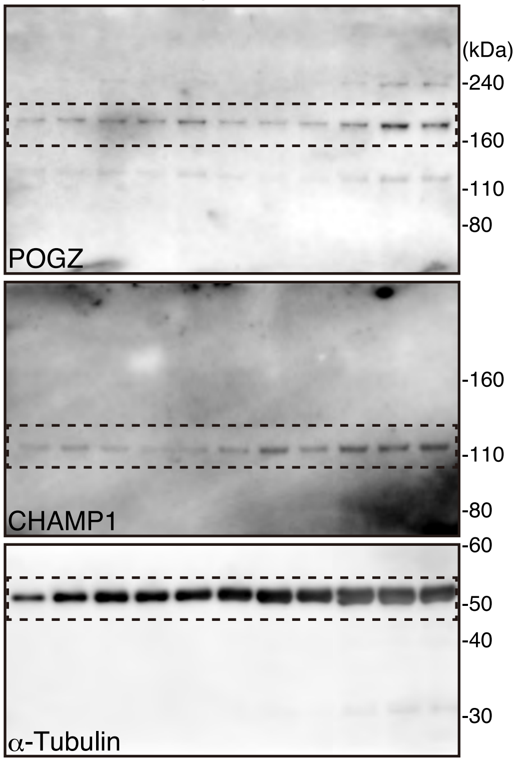

Figure 2B

sFigure 2E

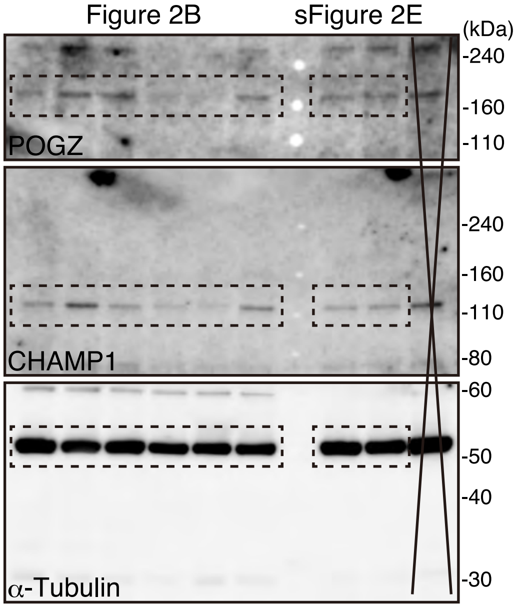

sFigure 7A

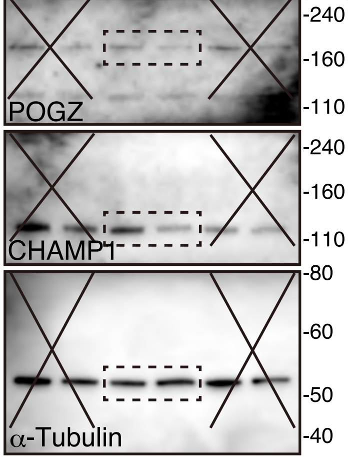

Supplementary Figure 12. Uncropped images of the Western blots shown in the figures.
